# Compensating for Electrode Polarization in Dielectric Spectroscopy Studies of Colloidal Suspensions: Theoretical Assessment of Existing Methods

**DOI:** 10.3389/fchem.2016.00030

**Published:** 2016-07-19

**Authors:** Claire Chassagne, Emmanuelle Dubois, María L. Jiménez, J. P. M van der Ploeg, Jan van Turnhout

**Affiliations:** ^1^Environmental Fluid Mechanics, Faculty of Civil Engineering and Geosciences, Delft University of TechnologyDelft, Netherlands; ^2^Laboratoire PHENIX, Centre National de la Recherche Scientifique, Sorbonne Universités, UPMC Université Paris 06Paris, France; ^3^Departamento de Física Aplicada, Universidad de GranadaGranada, Spain; ^4^Formerly affiliated with Leiden Institute of Chemistry, Leiden UniversityRA Leiden, Netherlands; ^5^Department of Chemical Engineering, Delft University of TechnologyDelft, Netherlands

**Keywords:** colloidal suspension, complex conductivity and permittivity, electrode polarization

## Abstract

Dielectric spectroscopy can be used to determine the dipole moment of colloidal particles from which important interfacial electrokinetic properties, for instance their zeta potential, can be deduced. Unfortunately, dielectric spectroscopy measurements are hampered by electrode polarization (EP). In this article, we review several procedures to compensate for this effect. First EP in electrolyte solutions is described: the complex conductivity is derived as function of frequency, for two cell geometries (planar and cylindrical) with blocking electrodes. The corresponding equivalent circuit for the electrolyte solution is given for each geometry. This equivalent circuit model is extended to suspensions. The complex conductivity of a suspension, in the presence of EP, is then calculated from the impedance. Different methods for compensating for EP are critically assessed, with the help of the theoretical findings. Their limit of validity is given in terms of characteristic frequencies. We can identify with one of these frequencies the frequency range within which data *uncorrected* for EP may be used to assess the dipole moment of colloidal particles. In order to extract this dipole moment from the measured data, two methods are reviewed: one is based on the use of existing models for the complex conductivity of suspensions, the other is the logarithmic derivative method. An extension to multiple relaxations of the logarithmic derivative method is proposed.

## 1. Introduction

Dielectric spectroscopy is a powerful tool to determine the electrokinetic properties of suspensions of nano- or microparticles as it can probe the suspension's response as function of the applied electric field frequency. Interfacial properties such as zeta potential and Stern layer conductance can then be derived by analyzing the dielectric spectra of the suspensions (Grosse et al., 1998; Delgado, 2002; Hollingsworth and Saville, 2003; Ohshima, 2006; Chassagne et al., 2009). It was shown recently that fitting simultaneously the dielectric spectrum of a suspension and the electrophoretic velocity of the particles composing this suspension can provide a unique set of parameters when a Stern layer conductance is necessary to fit the data in addition to the zeta potential (Chassagne et al., 2009). Most experiments on colloidal suspensions are performed in the “low frequency” regime, i.e., below 1 MHz, where the typical dispersions emerge that are associated with the colloidal particle and its double layer. Studies at higher frequencies (well above 1 MHz, see for instance Kaatze and Feldman, 2006) will not be addressed.

**Figure 1:**
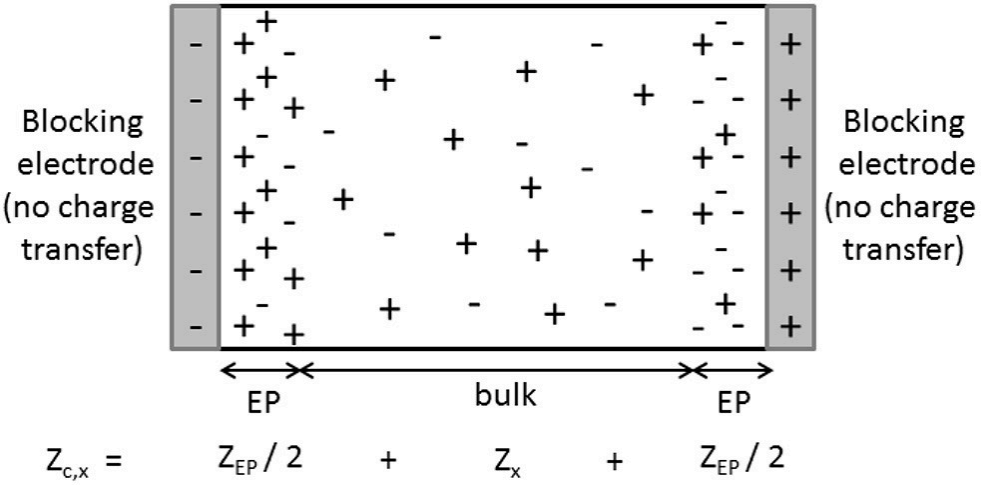
**Schematic representation of the distribution of particles (ions, colloidal particles) at a given time, when an AC electric field is applied to a solution (***x*** = ***e***) or suspension (***x*** = ***s***)**. Due to the blocking nature of the electrodes, ions and colloidal particles build-up close to the electrodes at low frequencies. The equivalent impedance of the cell ${\tilde{Z}}_{c,x}$ is then made up of two contributions: the wanted “true” impedance ${\tilde{Z}}_{x}$ of the bulk of the investigated solution or suspension and the contribution due to the polarization of the electrodes ${\tilde{Z}}_{EP}.$

The determination of dielectric spectra is based on measurements of the complex conductivity of the suspension ${\tilde{K}}_{s}$ (or equivalently the complex dielectric response ${\tilde{\varepsilon }}_{s}$) as a function of frequency. This is done by measuring the complex impedance ${\tilde{Z}}_{s}$ of a suspension contained in a cell with (generally two) electrodes. Although it is in principle a simple measurement, the proper determination of ${\tilde{Z}}_{s}$ is difficult due to several unwanted effects. Two types of effects are distinguished. One type of effect originates from the non-ideality of the experimental set-ups. Because of the presence of electrical circuits, wires, external components, so-called “stray impedances” will arise and have to be accounted for when the impedance of the cell, in which the suspension is inbedded, is measured. This type of effect will not be discussed here. Another type of unwanted effect stems from the distribution of the charged species in the measurement cell when an electric field is applied. It is the so-called “electrode polarization” (EP) that typically occurs at low frequencies, mostly below 10–100 kHz (Barsoukov and Macdonald, 2005). EP originates from the fact that at low frequencies ions are able to build up close to the electrodes, contributing with a large additional capacitance to the impedance ${\tilde{Z}}_{c,s}$ of the suspension, see Figure 1. The impedance ${\tilde{Z}}_{c,s}$ of the suspension measured in the cell (cleaned from all stray impedances that might have to be accounted for) can be seen as the sum of two impedances, the sought one from the bulk of the suspension ${\tilde{Z}}_{s}$ (“true” impedance) and an unwanted one due to EP, ${\tilde{Z}}_{EP}$:

$${\tilde{Z}}_{c,s}={\tilde{Z}}_{s}+{\tilde{Z}}_{EP}$$

As Hollingsworth (2013) recently pointed out, EP has been studied extensively. The impedance caused by the electrodes was first discussed by Kohlrausch around 1874 as a disturbance in conductivity measurements of electrolyte solutions. Theoretical work was performed by Warburg, Mandel, and Buck to name a few, see for instance Barsoukov and Macdonald (2005), Buck (1969), and van der Touw and Mandel (1971). Analytical expressions for the impedance by EP can be obtained by solving the set of relevant equations (Nernst-Planck, conservation of ions and Poisson). In most works (including the present article), the electrodes are assumed to be ideally polarizable, implying that faradaic reactions are negligible and hence that there is no charge transfer at the electrodes (blocking electrodes). We refer in particular to the work done in Hollingsworth and Saville (2003), Buck (1969), Cirkel et al. (1997), Chassagne et al. (2002), Chassagne et al. (2003) that will be used in this article. Theoretical investigation of electrode polarization with non-zero zeta potential at the electrodes has been performed numerically by White et al. (DeLacey and White, 1982), perturbation expansions have been done by Gunning et al. (1995) and semi-analytical solutions provided by Scott et al. (2000a,b).

The measurement of the complex dielectric response of colloids is hampered seriously by EP due to the fact that the characteristic relaxation frequencies associated with the properties of the colloids show up in the low-frequency regime too. It is therefore important to be able to devise a suitable method to remove or minimize the strong EP contribution to the measured signal, so as to assess ${\tilde{Z}}_{s}$. Recent reviews of the existing methods have been given by Kaatze et al. (Kaatze and Feldman, 2006; Grosse and Delgado, 2010; Ishai et al., 2013). The present article is intended to give insight in these different methods and in particular check their range of validity, from a theoretical perspective. Characteristic frequencies are derived. We show in particular that one of these frequencies enables us to identify those cases where data *uncorrected* for EP can be used to assess ${\tilde{Z}}_{s}$.

From ${\tilde{Z}}_{s},$ the complex conductivity ${\tilde{K}}_{s}$ of the suspension can be directly derived. This conductivity is related to the interfacial properties of the colloidal particles such as zeta potential and Stern layer conductance via the dipole moments of the polarized particles and their double layers. This dipole moment is represented by the dipolar coefficient $\tilde{\beta }$ that can be modeled either analytically or numerically, see e.g., Chassagne and Bedeaux (2008), Mangelsdorf and White (1990), and Minor et al. (1998). We will show, using the analytical model presented in Supplementary Material 1 as an example, how such a model can be used to find the interfacial properties of the particles by fitting data uncorrected for EP. In a next section, we will show how an extended logarithmic derivative method allows the evaluation of $\tilde{\beta }$ from uncorrected data. In the case the data should be corrected for EP, one of the different methods to account for it should be applied first.

### 1.1. Outline of the article

In Section 2, the purpose of dielectric spectroscopy in the context of colloids will be outlined. The complex conductivity ${\tilde{K}}_{s}$ of colloidal suspensions will be given as function of the complex dipolar coefficient $\tilde{\beta }$ as basic unknown. It is this quantity that characterizes important particles properties such as zeta potential and Stern layer conductances. Before discussing methods to find ${\tilde{K}}_{s}$ (and subsequently $\tilde{\beta }$) theoretical derivations are recalled: in Section 3, a description of EP for blocking electrodes will be given. This theory is an extension of previous ones (Buck, 1969; Cirkel et al., 1997; Chassagne et al., 2002, 2003), and is valid for the whole frequency range of interest and arbitrary mobilities and valences of ions. Two types of electrodes will be considered: planar and cylindrical, which correspond to the geometries most frequently used. Important results of the derivations given in Section 3 will be needed in Section 4. The impedance of the electrolyte (or suspension) is often represented as an equivalent circuit consisting of the combination of resistances and capacitances (Buck, 1969; Barsoukov and Macdonald, 2005). This formalism will be discussed in Section 4. It will in particular enable us to model the response of a colloidal suspension in the presence of EP. In Section 5, several methods to (a) correct for EP and (b) extract $\tilde{\beta }$ from the data will be presented and discussed. The Supplementary Material sections give details about: the mathematical derivations of some of the basic formulas reviewed, relevant aspects of the equivalent circuit and of the spatial profile of the alternating electric field within an electrolytic solution.

### 1.2. Variable definitions

${\tilde{Z}}_{c,x}$ is the impedance of the measurement cell filled with a suspension (*x* = *s*), an electrolyte solution (*x* = *e*) or a reference electrolyte solution (*x* = *r*) (all in the presence of electrode polarization).
${\tilde{Z}}_{e}$ is the “true” impedance of the electrolyte solution (without electrode polarization).
${\tilde{Z}}_{s}$ is the “true” impedance of the suspension (without electrode polarization).
${\tilde{Z}}_{r}$ is the “true” impedance of the reference electrolyte solution (without electrode polarization).
${\tilde{Z}}_{EP}$ is the impedance accounting for electrode polarization (${\tilde{Z}}_{EP}={\tilde{Z}}_{c,x}-{\tilde{Z}}_{x}$).

These subscripts apply accordingly to the related complex conductivities and complex permittivities ($\tilde{K}$, $\tilde{\varepsilon }$).

## 2. Link between measurement and particle's interfacial properties

When an alternating electric field *E*(ω) is applied to a colloidal suspension, the particles and their double layers become polarized. The charged particles will oscillate in the AC field as will the ions in the double layers, but with two different characteristic times. Such a combination of events usually produces a rich dielectric loss spectrum.

This interesting though complicated relaxation behavior can best be described by the complex dipolar coefficient $\tilde{\beta }\left(\omega \right)$ which is associated with the (complex) dipole moment of a single colloidal particle surrounded by its double layer. This dipole moment is generated by the polarization of the core material of the colloidal particle, and the anisotropic charge distributions of particle and ions in space and time, due to their respective movements. For a spherical colloidal particle of radius *a* the dipole coefficient $\tilde{\beta }$ is defined by:

(1) $$\tilde{P}\left(\omega \right)=4\pi \varepsilon_{0}\varepsilon_{e}a^{3}\tilde{\beta }\left(\omega \right)E\left(\omega \right)$$

where $\tilde{P}$ is the particle's dipole moment, ε_*e*_ the relative dielectric permittivity of the solvent (generally water) and ε_0_ the permittivity of vacuum. Since this quantity incorporates all polarization mechanisms possible, we can gather with dielectric spectroscopy important information from $\tilde{\beta }$ about the interfacial properties of the particles, in particular the zeta potential and Stern layer conductances of the particle (DeLacey and White, 1981; Delgado, 2002; Ohshima, 2006; Chassagne and Bedeaux, 2008). A general expression of $\tilde{\beta }$ can be found in Supplementary Material 1, see also Chassagne and Bedeaux (2008), Mangelsdorf and White (1990), and Minor et al. (1998). A discussion about the important characteristic frequencies associated with the system can be found in Grosse and Shilov et al.

The relation between dipolar coefficient and complex conductivity ${\tilde{K}}_{s}\left(\omega \right)$ of a suspension with low volume fraction ϕ of the dispersed particles is given by Delgado (2002):

(2) $$\begin{array}{l} {\tilde{K}}_{s}\left(\omega \right)={\tilde{K}}_{e}\left(\omega \right)\left(1+3\phi \tilde{\beta }\left(\omega \right)\right)\\ \;=K_{s}\left(\omega \right)+i\omega \varepsilon_{s}\left(\omega \right)\varepsilon_{0} \end{array}$$

where ε_*s*_(ω) is the real part of the permittivity of the suspension and *K*_*s*_(ω) the real part of its conductivity. A precise derivation of this relation can be found in Grosse. The complex conductivity of the suspending electrolyte is given by

(3) $${\tilde{K}}_{e}\left(\omega \right)=K_{e}+i\omega \varepsilon_{0}\varepsilon_{e}$$

where *K*_*e*_ is the Ohmic conductivity of the electrolyte solution, defined explicitly later in Equation (28). *K*_*e*_ equals in principle the conductivity obtained in an ideal DC conductivity experiment. We assume both *K*_*e*_ and ε_*e*_ to be frequency-independent. This hypothesis is justified for the frequencies used. In Chassagne and Bedeaux (2008), the notation (*K*_1_, ε_1_) was used instead of (*K*_*e*_, ε_*e*_) to express the conductivity and relative dielectric permittivity of the electrolyte. Instead of the complex conductivity $\tilde{K}\left(\omega \right)$ for the suspension and the electrolyte we can equally well use the complex permittivity $\tilde{\varepsilon }\left(\omega \right)$ defined by:

(4) $$\tilde{\varepsilon }\left(\omega \right)\equiv \frac{\tilde{K}\left(\omega \right)}{i\omega \varepsilon_{0}}$$

It then follows that:

(5) $$\begin{array}{l} {\tilde{\varepsilon }}_{s}\left(\omega \right)={\tilde{\varepsilon }}_{e}\left(\omega \right)(1+3\phi \tilde{\beta }\left(\omega \right))\\ \;=\varepsilon_{s}\left(\omega \right)-i\frac{K_{s}\left(\omega \right)}{\omega \varepsilon_{0}} \end{array}$$

Some authors prefer to overlook the interrelation in Equations (2) and (5) between the components of $\tilde{K}$ and $\tilde{\varepsilon }$ and simply write:

(6) $$\begin{array}{l} \tilde{K}\left(\omega \right)=K\prime \left(\omega \right)+iK\prime \prime \left(\omega \right)\\ \tilde{\varepsilon }\left(\omega \right)=\varepsilon \prime \left(\omega \right)-i\varepsilon \prime \prime \left(\omega \right) \end{array}$$

with the real parts denoted by a single prime and the imaginary parts by a double prime. From which we can deduce that:

(7) $$\begin{array}{l} K_{s}^{\prime }\left(\omega \right)=K_{s}\left(\omega \right)\text{ and }\varepsilon_{s}^{\prime }\left(\omega \right)=\varepsilon_{s}\left(\omega \right)\\ K_{s}^{\prime \prime }\left(\omega \right)=\omega \varepsilon_{s}\left(\omega \right)\varepsilon_{0}\text{ and }\varepsilon_{s}^{\prime \prime }\left(\omega \right)=\frac{K_{s}\left(\omega \right)}{\omega \varepsilon_{0}} \end{array}$$

Dielectric spectroscopy data are usually plotted in terms of dielectric and conductivity increments, that can be obtained from Equations (2) and (5) and are defined by:

(8) $$\begin{array}{l} \Delta \varepsilon_{s}\left(\omega \right)=\frac{\varepsilon_{s}\left(\omega \right)-\varepsilon_{e}}{\phi }=3\;[\varepsilon_{e}\text{Re }\left(\tilde{\beta }\left(\omega \right)\right)+K_{e}\frac{\text{Im }\left(\tilde{\beta }\left(\omega \right)\right)}{\omega \varepsilon_{0}}]\\ \Delta K_{s}\left(\omega \right)=\frac{K_{s}\left(\omega \right)-K_{e}}{\phi }=3\;[K_{e}\text{Re }\left(\tilde{\beta }\left(\omega \right)\right)-\varepsilon_{0}\varepsilon_{e}\omega \text{Im}\left(\tilde{\beta }\left(\omega \right)\right)] \end{array}$$

As we are interested in the dipolar coefficient, it is convenient to write Equations (2) and (5) in terms of the real and imaginary parts of $\tilde{\beta }$:

(9) $$\begin{array}{l} \text{Re }\left(\tilde{\beta }\left(\omega \right)\right)=\frac{1}{3\phi }\frac{(K_{s}\left(\omega \right)-K_{e})/(\varepsilon_{0}\varepsilon_{e}\omega )+\varepsilon_{0}\left(\varepsilon_{s}\left(\omega \right)-\varepsilon_{e}\right)\omega /K_{e}}{\left(\varepsilon_{0}\varepsilon_{e}\omega )/K_{e}+K_{e}/(\varepsilon_{0}\varepsilon_{e}\omega \right)}\\ \text{Im }\left(\tilde{\beta }\left(\omega \right)\right)=\frac{1}{3\phi }\frac{-(K_{s}\left(\omega \right)-K_{e})/K_{e}+\left(\varepsilon_{s}\left(\omega \right)-\varepsilon_{e}\right)\omega /\varepsilon_{e}}{\left(\varepsilon_{0}\varepsilon_{e}\omega )/K_{e}+K_{e}/(\varepsilon_{0}\varepsilon_{e}\omega \right)} \end{array}$$

These two relations enable us to understand an additional source for the errors that can be made in the evaluation of the dipole coefficient. Indeed, at low frequencies, where the magnitude |$\tilde{\beta }$| of the dipolar coefficient is usually the largest, we obtain:

(10) $$\begin{array}{l} \text{Re }\left(\tilde{\beta }\left(\omega \to 0\right)\right)=\frac{K_{s}\left(\omega \to 0\right)-K_{e}}{3K_{e}\phi }\\ \text{Im }\left(\tilde{\beta }\left(\omega \to 0\right)\right)=0 \end{array}$$

As the conductivity of the (dilute) suspension is in general very close to the conductivity of the suspending electrolyte, *K*_*s*_ ≃ *K*_*e*_, a small inaccuracy in the measurement of *K*_*s*_ and/or *K*_*e*_ will give rise to a large change in *Re*(β) and consequently |$\tilde{\beta }$|.

In practice ${\tilde{K}}_{s}$ (or equivently ${\tilde{\varepsilon }}_{s}$) are not measured. The measured impedance of the suspension contains interfering impedances that have to be corrected for. In the introduction we distinguished between two types of effects: effects that originate from the actual electrical connections, leading to stray impedances, and effects coming from the ionic charge distribution in the cell close to the electrodes, leading to Electrode Polarization (EP) impedances. In the present article, only EP impedances will be discussed. This implies that we assume that we have properly compensated for stray impedances and that we have access to the complex impedance ${\tilde{Z}}_{c,s}$. This impedance ${\tilde{Z}}_{c,s}$ is the impedance of the cell filled with the suspension. ${\tilde{Z}}_{c,s}$ contains the EP contribution ${\tilde{Z}}_{EP},$ but no stray impedances contributions, and the contribution of the bulk of the suspension, i.e., ${\tilde{Z}}_{s}$ that we are looking for:

(11) $${\tilde{Z}}_{c,s}={\tilde{Z}}_{s}+{\tilde{Z}}_{EP}$$

The general relation between impedance and conductivity (for any subscript: *x* = *c, s*, *x* = *s*, *x* = *c, e* or *x* = *e*) is given by:

(12) $$1/{\tilde{Z}}_{x}=Ci\omega \varepsilon_{0}{\tilde{\varepsilon }}_{x}=C{\tilde{K}}_{x}=C\left(K_{x}+i\omega \varepsilon_{0}\varepsilon_{x}\right)$$

where *C* is a cell constant that depends on the geometry of the electrodes. This relation is only valid in the case that the electrodes are blocking. In case they are not, there would be a remaining DC conductivity at ω = 0. From Equation (12), it follows that for blocking electrodes ${\tilde{K}}_{c,x}\left(\omega =0\right)=K_{c,x}\left(\omega =0\right)+0\times i\varepsilon_{0}\varepsilon_{c,x}\left(\omega =0\right)=0$ and therefore *K*_*c, x*_(ω = 0) = 0.

The cell constant *C* is defined in Supplementary Material 2 and derived in the corresponding sections for planar and cylindrical electrodes. We have:

(13) $$\begin{array}{l} C=\frac{S}{d}\;(\text{planar electrodes})\\ C=2\pi \;h\;(\text{cylindrical electrodes}) \end{array}$$

## 3. Complex permittivity of an electrolyte in presence of electrode polarization

In this section, the complex permittivity ${\tilde{\varepsilon }}_{c,e}\left(\omega \right)$ of an electrolyte solution, in the presence of electrode polarization, is analyzed for the planar electrode case. The important hypotheses used for the derivations are:

1 - In the absence of an applied voltage the electrodes are uncharged.

2 - No charge transfer takes place at the electrodes. These “ideally polarizable electrodes” can therefore be considered as capacitor plates.

3 - The electrodes are spaced sufficiently apart so that their respective EP does not influence each other, i.e., |λ_*c*_*d*| ≫ 1 (these variables are defined right below), a valid assumption for the experimental conditions encountered in impedance spectroscopy.

The solution is obtained from the set of relevant equations (Nernst-Planck, conservation of ions, Poisson) and appropriate boundary conditions. The mathematical details of the derivations for both planar and cylindrical electrodes can be found in Supplementary Material 2. The electrolyte response to the applied electric field frequency is shown to display the same characteristic features, besides geometrical aspects, in both cases.

We here recall the expressions of important variables defined in Supplementary Material 2, which are needed to evaluate the complex permittivities given below:

(14) $$D_{0}=\frac{z_{+}D_{+}-z_{-}D_{-}}{z_{+}-z_{-}}$$

(15) $$D_{n}=\frac{z_{+}-z_{-}}{z_{+}/D_{-}-z_{-}/D_{+}}$$

(16) $$D_{c}=\frac{z_{+}-z_{-}}{z_{+}/D_{+}-z_{-}/D_{-}}$$

(17) $$\lambda_{n}\left(\omega \right)={\left[\frac{\kappa^{2}}{2}\left(1-R\left(\omega \right)\right)+\frac{i\omega }{2}\left(\frac{1}{D_{+}}+\frac{1}{D_{-}}\right)\right]}^{1/2}$$

(18) $$\lambda_{c}\left(\omega \right)={\left[\frac{\kappa^{2}}{2}\left(1+R\left(\omega \right)\right)+\frac{i\omega }{2}\left(\frac{1}{D_{+}}+\frac{1}{D_{-}}\right)\right]}^{1/2}$$

with

(19) $$\begin{array}{l} R\left(\omega \right)\\ =\sqrt{1-\frac{\omega^{2}}{\kappa^{4}}{\left(\frac{1}{D_{+}}-\frac{1}{D_{-}}\right)}^{2}+\frac{2i\omega }{\kappa^{2}}\frac{\nu_{+}z_{+}^{2}-\nu_{-}z_{-}^{2}}{\nu_{+}z_{+}^{2}+\nu_{-}z_{-}^{2}}\left(\frac{1}{D_{+}}-\frac{1}{D_{-}}\right)} \end{array}$$

where:

**Table:** 

ν_*i*_	is the stoichiometric coefficient of ion *i* with *i* = +, −
*z_i_*	is the valence of ion *i*
*D_i_*	is the diffusion constant of ion *i*
ω	is the radial frequency of the applied electric field
κ^−1^	is the Debye length

Furthermore *d* is the distance between electrodes.

The diffusion coefficients of the ions can be obtained from their limiting conductivities $\Lambda_{i}^{\infty }$ (which can be found in Handbooks) from the relation:

(20) $$\Lambda_{i}^{\infty }=\left|z_{i}\right|D_{i}N_{a}e^{2}/(kT)$$

where *N*_*a*_ is Avogadro's number, *e* the electric elementary charge, *k* Boltzmann's constant and *T* the temperature. The Debye length can be obtained from the relation:

(21) $$\kappa^{2}=e^{2}n_{\infty }{\left(\varepsilon_{0}\varepsilon_{e}kT\right)}^{-1}\sum \nu_{i}z_{i}^{2}$$

where the ionic density is defined by

(22) $$n_{\infty }(m^{-3})=C_{s}(\text{mM})\times N_{a}$$

where *C*_*s*_ is the salt concentration in 10^−3^ mol/L (i.e., millimolar, mM).

**Figure 2:**
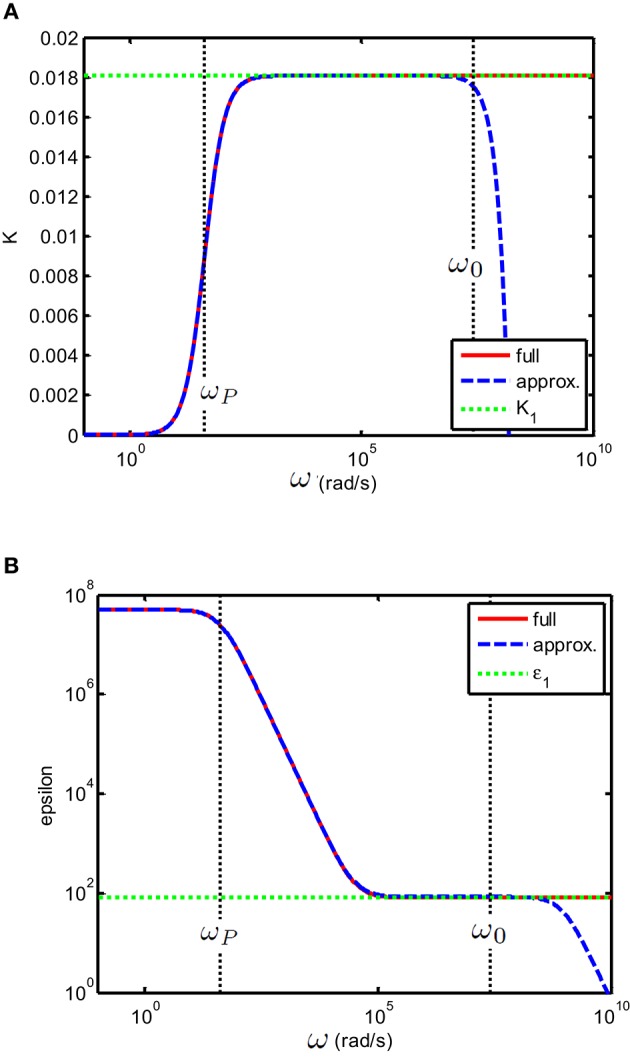
**(A)** Conductivity K(S/m) and **(B)** relative permittivity (epsilon) of a solution of divalent salt as function of the applied electric field frequency, similar to MgCl2, for which *D*_1_ = 1.4 × 10^−9^ m^2^/s and *D*_2_ = 2.0 × 10^−9^ m^2^ /s. The salt concentration is 0.5 mM. The electrodes are planar. The important characteristic frequencies associated with the system are $\omega_{0}=D_{0}\kappa^{2}$ and ω_*P*_ = 2κ*D*_0_/*d* where *d* = 10 × 10^−3^ m is the distance between the electrodes. The full solution found in this study, i.e., Equation (23) (red full line) is compared to the solution (blue dashed line) found in Chassagne et al. (2002), which was derived for the case ω ≪ ω_0_. As expected, the two solutions overlap for ω ≪ ω_0_. The green dotted line in the conductivity plot represents the theoretical conductivity, the value of which is given by Equation (29). The green dotted line in the epsilon plot represents the relative permittivity of water.

In Supplementary Material 2 it is shown that the measured complex permittivity of a binary electrolyte solution, in the case of planar electrodes is given by

(23) $$\begin{array}{l} \;{\tilde{\varepsilon }}_{c,e}\left(\omega \right)\\ =\varepsilon_{e}/[1-\frac{\kappa^{4}}{\left(\lambda_{c}^{2}-\lambda_{n}^{2}\right)}[\left(1+\frac{i\omega }{\kappa^{2}D_{c}}-\frac{\lambda_{n}^{2}}{\kappa^{2}}\right)\frac{1}{\lambda_{c}^{2}}\left[1-\frac{2}{\lambda_{c}d}\right]\\ \;-\left(1+\frac{i\omega }{\kappa^{2}D_{c}}-\frac{\lambda_{c}^{2}}{\kappa^{2}}\right)\frac{1}{\lambda_{n}^{2}}\left[1-\frac{2}{\lambda_{n}d}\right]]] \end{array}$$

for all frequencies, valences and ionic strengths. One can verify that for frequencies such that $\omega \ll D_{\pm }\kappa^{2}$, this expression corresponds to the expression found in Chassagne et al. (2002), which is also valid for all types of electrolytes but for frequencies restricted to $\omega \ll D_{\pm }\kappa^{2}.$ An illustration is given in Figure 2. The condition $\omega \ll D_{\pm }\kappa^{2}$ corresponds to the one encountered mostly in experiments. For these frequencies, one also finds that

(24) $$\lambda_{n}^{2}≃\frac{i\omega }{D_{n}}\text{ and }\lambda_{c}^{2}≃\kappa^{2}+\frac{i\omega }{D_{c}}$$

The lengthscale $\lambda_{c}^{-1}$ is related to the creation of the double layer close to the electrodes: for low frequencies $\left(\omega \ll D_{\pm }\kappa^{2}\right)$ one gets $\lambda_{c}^{-1}≃\kappa^{-1}$ and for high frequencies $\lambda_{c}^{-1}≃0$. The lengthscale $\lambda_{n}^{-1}$ is related to the ionic diffusion.

In the particular case where *D*_−_ = *D*_+_ = *D* which has been treated by several authors, see Cirkel et al. (1997), Buck (1969), and Hollingsworth and Saville (2003) one finds the simplifications:

(25) $$\begin{array}{l} {\tilde{\varepsilon }}_{c,e}\left(\omega \right)=\varepsilon_{e}/\left[1-{\left(\frac{\kappa }{\lambda_{c}}\right)}^{2}\left[1-\frac{2}{\lambda_{c}d}\right]\right]\\ \text{with }\lambda_{c}=\sqrt{\kappa^{2}+i\omega /D} \end{array}$$

Equation (25) is for example the expression found by Cirkel et al. [their Equation (8) in Cirkel et al., 1997]. Explicit expressions for the real and imaginary part of ${\tilde{\varepsilon }}_{c,e}$ are given in Supplementary Material 4. There, we also make a comparison with the work done by Kang et al. (Kang and Dhont, 2010) on the in and out phase component of the alternating electric field. Since we have made the hypothesis that λ_*c*_*d* ≫ 1 we can safely assume that *tanh*(λ_*c*_*d*/2) ≃ 1 and Equation (25) is also in agreement with Equation (25) of Hollingsworth et al. in (Hollingsworth and Saville, 2003), who restricted their calculations to 1:1 electrolytes (for which *z*_+_ = −*z*_−_ = 1).

Two important relaxations frequencies are found from the analysis of Equation (23):

(26) $$\begin{array}{l} \omega_{P}=\frac{2\kappa D_{0}}{d}≃\frac{2\kappa D_{\pm }}{d}\\ \omega_{0}=D_{0}\kappa^{2}≃D_{\pm }\kappa^{2} \end{array}$$

The frequency ω_*P*_ corresponds to the frequency below which charges can fully build-up a double layer close to the blocking electrodes due to the application of the alternating electric field. Below ω_*P*_ and beyond the double layers, the bulk electric field is consequently zero. For ω ≪ ω_*P*_ we get:

(27) $$\begin{array}{l} \varepsilon_{c,e}≃\varepsilon_{e}\frac{\kappa d}{2}\\ K_{c,e}≃\varepsilon_{e}\varepsilon_{0}{\left(\frac{\kappa d}{2}\right)}^{2}\frac{\omega^{2}}{\kappa^{2}D_{0}}≃0 \end{array}$$

The conductivity *K*_*c, e*_ is in good approximation zero for low frequencies because the electrodes are blocking. No charge transfer is possible and the ions are all accumulated close to the electrodes.

The Maxwell-Wagner frequency ω_0_ represents the frequency below which charges can be dissipated in the system. For ω_*P*_ ≪ ω ≪ ω_0_ we get:

(28) $$\begin{array}{l} \varepsilon_{c,e}\left(\omega \right)≃\varepsilon_{e}/\left[\frac{\kappa d}{2}{\left(\frac{\omega }{\kappa^{2}D_{0}}\right)}^{2}\right]\\ K_{c,e}≃\varepsilon_{e}\varepsilon_{0}\kappa^{2}D_{0}\equiv K_{e} \end{array}$$

As pointed out in recent discussions (Grosse and Delgado, 2013; Hollingsworth, 2013), we note that ε_*c, e*_ indeed scales as ω^−2^ in this frequency range. The conductivity in this frequency range is equal to the theoretical conductivity *K*_*e*_ of the electrolyte solution. We didn't include ionic interactions, which would have modified the value of *K*_*e*_ presented here. This implies in particular that we consider electrolytes at low ionic strength, for which the conductivity varies linearly with the ionic strength ($K_{e}\propto \kappa^{2}\propto C_{s}$ where *C*_*s*_ is the salt concentration). Above ω_0_ no double layer can be established at the electrodes and the electric field in the cell is everywhere equal to the applied one. For ω ≫ ω_0_ we get:

(29) $$\begin{array}{l} \varepsilon_{c,e}≃\varepsilon_{e}\\ K_{c,e}≃\varepsilon_{0}\varepsilon_{e}\kappa^{2}D_{0}\equiv K_{e} \end{array}$$

The permittivity of the electrolyte solution has reduced to the one of water (we do not consider the frequency-dependence of ε_*e*_) and the conductivity is equal to the usual conductivity of the electrolyte solution since we have not considered inertial effects that might slow down the ions at high frequencies. The relaxation frequency ω_0_ is defined by the frequency above which ε_0_ε_*e*_ω > *K*_*e*_ i.e., $\omega_{0}=K_{e}/\left(\varepsilon_{0}\varepsilon_{e}\right)=D_{0}\kappa^{2}.$ It can be verified that for $\omega \gg D_{\pm }\kappa^{2}$ the permittivity (ε_*c,e*_ − ε_*e*_) scales as ω^−3/2^ (Cirkel et al., 1997; Hollingsworth, 2013). However, this dependence can not be observed. If one takes an electrolyte such that *D*_−_ = *D*_+_ = *D* the expansion of Equation (25) reads:

(30) $$\varepsilon_{c,e}\left(\omega \right)≃\varepsilon_{e}\left[1+\frac{1}{\sqrt{2}}\frac{2}{\kappa d}{\left(\frac{\kappa^{2}D}{\omega }\right)}^{3/2}\right]$$

Even under extremely good conditions such that, for instance ω ≃ κ^2^*D*, for an electrode spacing of 1 mm and a very low ionic strength of 0.1 mM NaCl, one finds $\varepsilon_{c,e}≃\varepsilon_{e}\left[1+10^{-5}\right]$ and the correcting term is virtually undetectable.

Indicative values of ω_*P*_ and ω_0_ are given in Table 1.

**Table 1:** **Values of ω_***P***_ and ω_**0**_ as given by Equation (26), using a diffusion coefficient of $D_{0}=2.10^{-9}$ m^**2**^/s at room temperature, for 3 salt concentrations**.

***C_s_* (mM)**	**0.1**	**1**	**10**
ω_*P*_(*d* = 1 *mm*, rad/s)	130	415	1300
ω_*P*_(*d* = 10 *mm*, rad/s)	13	41	130
ω_0_(rad/s)	2.10^6^	2.10^7^	2.10^8^

## 4. Equivalent circuits

**Figure 3:**
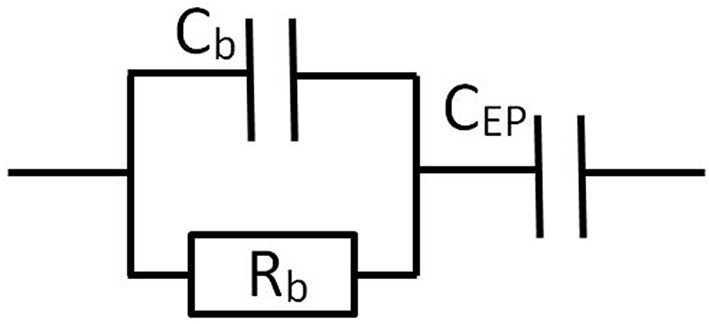
**Equivalent circuit representation for a cell containing a solution or suspension, with blocking electrodes**.

Traditionally Dielectric Spectroscopy measurements have been described by equivalent electrical circuits (Barsoukov and Macdonald, 2005). A comparison between the results found from equivalent circuits and those found from solving the electrokinetic set of equations has been performed in specific cases (Buck, 1969; Barsoukov and Macdonald, 2005). We are going to show that the description of the system (any electrolyte solution or colloidal suspension) using equivalent circuits is strictly equivalent to the results found in the previous section, for all frequencies and cell geometries (planar and cylindrical). We will start with the description of electrolyte solutions. The results for colloids will be given in the following section. The solution can be described by a resistance *R*_*b*_ in parallel with a capacitor *C*_*b*_. The electrode polarization can be seen as an additional capacitor *C*_*EP*_ in series with the equivalent circuit of the solution, see Figure 3.

The total impedance of the system ${\tilde{Z}}_{c}$ (including electrode polarization) can be evaluated from the relations:

$${\tilde{Z}}_{c}={\tilde{Z}}_{s}+\frac{1}{i\omega C_{EP}}$$

(31) $$\frac{1}{{\tilde{Z}}_{s}}=\frac{1}{R_{b}}+i\omega C_{b}$$

The general expression for ${\tilde{Z}}_{c}$ is given in Supplementary Material 2. From that expression, one can verify that for ω_0_ ≫ ω ≫ ω_*P*_ one gets

(32) $$\frac{1}{{\tilde{Z}}_{c}}≃\frac{1}{R_{b}}+i\omega \left[\frac{1}{\omega^{2}R_{b}^{2}C_{EP}}+C_{b}\right]$$

Schwan (1992) found this result experimentally in the case of a concentrated suspension of blood cells. Like many experimentalists, he expresses his complex measured impedance in terms of a resistance and capacitance in series i.e.,

(33) $$\frac{1}{{\tilde{Z}}_{c}}=\frac{1}{R}+i\omega C$$

and defines:

$$R=R_{b}$$

(34) $$C=C_{b}+\frac{1}{\omega^{2}R_{b}^{2}C_{EP}}$$

In Figure 5 of Schwan (1992), it is shown that $C≃1/\left(\omega^{2}R_{b}^{2}C_{EP}\right)$ for low frequencies and that *C* ≃ *C*_*b*_ at high frequencies. As EP only contributes as a capacitance, it implies that EP only affects *C* and not *R*. In other words, in the frequency range of interest (ω_0_ ≫ ω ≫ ω_*P*_) only the permittivity is affected by EP, not the conductivity, which reduces to the bulk conductivity. This is consistent with the results of Section 3.

### 4.1. Electrolyte solutions

In Supplementary Material 3 we describe how we obtain the expressions for *C*_*EP*_, *C*_*b*_, and *R*_*b*_ from the mathematical equivalence with the analytical results of Section 3. In the case of planar electrodes, we find:

$$C_{EP}=\frac{S}{d}\varepsilon_{0}\varepsilon_{e}\frac{\kappa d}{2}$$

$$C_{b}=\frac{S}{d}\varepsilon_{e}\varepsilon_{0}$$

(35) $$R_{b}=\frac{d}{S}\frac{1}{K_{e}}$$

These values were also found by Buck (1969) in the case of a z-z electrolyte. One can verify that the characteristic frequencies found in Supplementary Material 3 can be related to the ones found in the Planar electrodes section by:

$$\omega_{P}=\frac{1}{R_{b}C_{EP}}=\frac{2\kappa D_{0}}{d}$$

$$\omega_{0}=\frac{1}{R_{b}C_{b}}=D_{0}\kappa^{2}$$

The signification of the frequencies (specified in Supplementary Material 2) can now be linked to circuit elements: as expected ω_*P*_ depends on the capacitance *C*_*EP*_ associated to electrode polarization, whereas ω_0_ depends on *C*_*b*_ associated to the bulk permittivity. Note that the “2” that appears in the right hand side part of the relation for ω_*P*_ can be seen as resulting from the fact that there are two electrodes in the system: each electrode contributes with a capacitance *Sε*_0_ε_*e*_κ and summing two capacitances in series yields *C*_*EP*_ = *Sε*_0_ε_*e*_κ/2 as in Equation (35). Some authors therefore prefer to use an alternative equivalent circuit : instead of the one represented in Figure 3, where one capacitor of capacitance *C*_*EP*_ = *Sε*_0_ε_*e*_κ/2 was used, one can have a circuit with two capacitors, each of capacitance *C*_*EP*_ = *Sε*_0_ε_*e*_κ, placed on each side of the ${\tilde{Z}}_{s}$ element, as was sketched in Figure 1.

Following the same procedure for the cylindrical electrodes (not detailed here), one finds the equivalence, using the circuit represented in Figure 3:

$$C_{EP}=2\pi \;h\frac{\varepsilon_{0}\varepsilon_{e}\kappa }{\left(R_{1}^{-1}+R_{2}^{-1}\right)}$$

$$C_{b}=2\pi \;h\frac{\varepsilon_{0}\varepsilon_{e}}{\text{ln}\left(R_{2}/R_{1}\right)}$$

(36) $$R_{b}=\frac{1}{2\pi \;h}\frac{\ln\left(R_{2}/R_{1}\right)}{K_{e}}$$

The analytical expressions of Section 3 and the equivalent circuit expressions give the same results for all frequencies, ionic strengths, and type of salts (not shown). Buck (1969) also derived an equivalent circuit in the case of spherical concentric electrodes for z-z electrolytes. We refer to him for this geometry which will not be further discussed, as most experiments nowadays are performed with either planar or cylindrical electrodes.

### 4.2. Colloidal suspensions

For the sake of argument, we will consider planar electrodes. Instead of a simple electrolyte solution, as studied in the previous section, we now like to find an equivalent circuit in the case a complex electrolyte solution such as a colloidal suspension. We make the hypothesis that the equivalent circuit can be represented as the one given in Figure 3: we would like that when the concentration of colloidal particles goes to zero, one would find again Equation (35). The equivalent circuit elements are taken to be:

$$C_{EP}\left(\omega \right)=\frac{S}{d}\varepsilon_{0}\varepsilon^{*}\left(\omega \right)\frac{\kappa d}{2}$$

$$C_{b}\left(\omega \right)=\frac{S}{d}\varepsilon_{s}\left(\omega \right)\varepsilon_{0}$$

(37) $$R_{b}\left(\omega \right)=\frac{d}{S}\frac{1}{K_{s}\left(\omega \right)}$$

**Figure 4:**
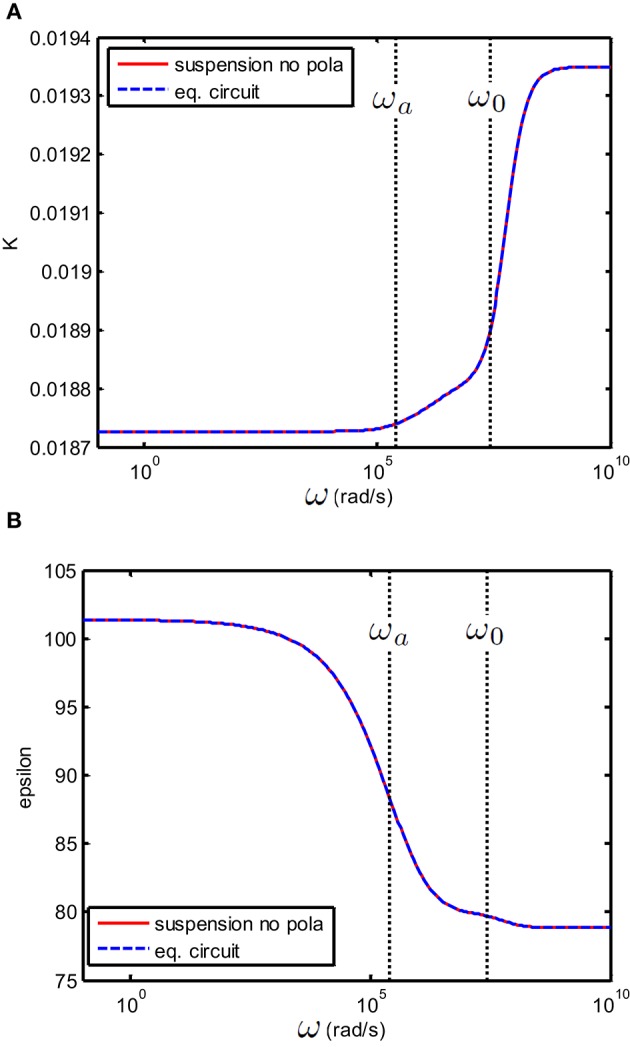
**(A)** Conductivity K(S/m) and **(B)** relative permittivity (epsilon) as function of frequency of a suspension of 100 nm colloidal spheres (ϕ = 1%, *eζ*/*kT* = 4) in a 1 mM electrolyte solution of monovalent salt solution for which $D_{1}=2\times 10^{-9}$ m^2^/s and $D_{2}=3\times 10^{-9}$ m^2^/s. Red curve: the suspension in the absence of electrode polarization, corresponding to ε_*s*_ and *K*_*s*_ from Equation (8). Dashed blue curve: the equivalent circuit model corresponds to the theoretical prediction provided that one takes *R*_*b*_ = 1/*K*_*s*_, *C*_*b*_ = ε_0_ε_*s*_ and *C*_*EP*_ = 0.

Note that in the general case the circuit elements now become frequency-dependent: even though the *R* and *C* in Equation (37) have the dimensions of resistance and capacitance, they represent no “real” resistor and capacitors, and no “real” electronic equivalent circuit can be made of them. This was not the case for electrolyte solutions, where all *R* and *C* were frequency-independent, and which could therefore be substituted by “real” resistor and capacitors. The equivalent circuit would then reproduce the behavior of the electrolyte between blocking electrodes. The expressions for ε_*s*_ and *K*_*s*_ are given in Equation (8). An important check for the validity of the proposed equivalent circuit is that in the absence of electrode polarization, the analytical expression for ${\tilde{K}}_{s}$ and the equivalent circuit expression should give the same results: this is illustrated in Figure 4. This is not surprising, as one can see by comparing Equations (12) and (31).

If there are no colloidal particles, we get from Equation (5) that ${\tilde{\varepsilon }}_{s}\left(\omega \right)={\tilde{\varepsilon }}_{e}\left(\omega \right)$ and we indeed find again Equation (35), provided that the unknown ε^*^ equals ε_*e*_. The permittivity ε^*^ is clearly linked to the EP phenomena by construction. One could argue that EP originates mainly from the contribution of the ions and not that of the colloidal particles. This implies that the dielectric permittivity ε^*^ in the small slab of liquid adjacent to the electrodes should be equal to ε_*e*_ whether in the presence or not of colloidal particles. If the colloidal particles contribute to ε^*^, ε^*^ should be close to ε_*s*_(ω). The relation between ε_*s*_ and ε_*e*_ is given by Equation (8), and these permittivities do not differ much, especially not at low volume fractions. However, even a small difference in permittivities will prevent that the subtraction method is applicable when $\varepsilon^{*}\neq \varepsilon_{e}$ (See section 5). We note that both *C*_*b*_ and *R*_*b*_ depend on *d*, whereas *C*_*EP*_ does not. This fact will allow to account for EP by the variable separation method (See section 5).

**Figure 5:**
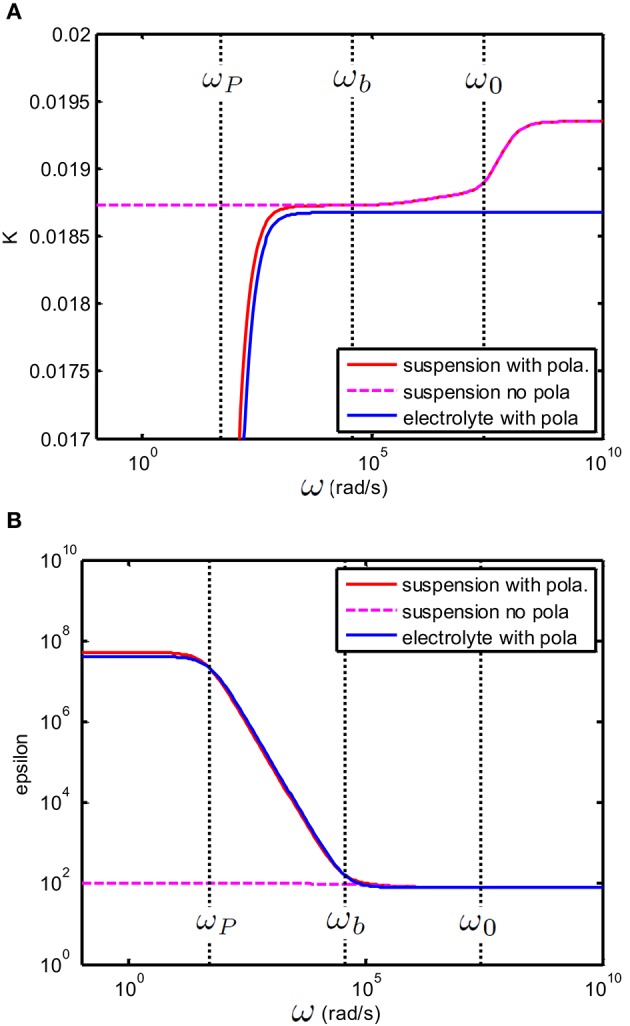
**(A)** Conductivity K(S/m) and **(B)**: relative permittivity (epsilon) as function of frequency. Suspension with same properties as the one given in Figure 4. The suspending electrolyte solution (blue line) and suspension (red line) in the presence of electrode polarization. The magenta dashed line corresponds to the solution found in Figure 4 for no EP. The equivalent circuit of the suspension in the presence of electrode polarization was constructed by taking *R*_*b*_ = 1/*K*_*s*_, *C*_*b*_ = ε_0_ε_*s*_ and *C*_*EP*_ = ε_0_ε_*s*_κ*d*/2 with *d* = 10 mm.

In Figure 4, we have shown the case where *C*_*EP*_ = 0 (no electrode polarization). Two relaxations features can be observed that are related to the characteristic frequencies associated with the polarization of the particle and its double layer. The double layer around a colloidal particle and the double layer at the electrodes have the same relaxation frequency ω_0_ and the frequency associated with the relaxation of the particle is given by $\omega_{a}=D_{0}/a^{2}$ where *a* is the radius of the colloidal particle and *D*_0_ is given in Equation (14). If we take electrode polarization into account, the dielectric permittivity at low frequency is 10^6^ times larger than that without electrode polarization. This is illustrated in Figure 5. In this example we have used $\varepsilon^{*}=\varepsilon_{s}$.

In the frequency range of interest, i.e., for ω_0_ ≫ ω ≫ ω_*P*_ Equation (32) holds. A new frequency can be defined by

(38) $$\omega_{b}=1/\left(R_{b}\sqrt{C_{b}C_{EP}}\right)=\sqrt{\omega_{0}\omega_{P}}$$

This frequency is discussed in Supplementary Material 4. One can verify from Equation (32) that for ω ≫ ω_*b*_ the influence of electrode polarization can be neglected and

(39) $$\frac{1}{{\tilde{Z}}_{c}\left(\omega \right)}≃i\omega C_{b}+\frac{1}{R_{b}}$$

**Figure 6:**
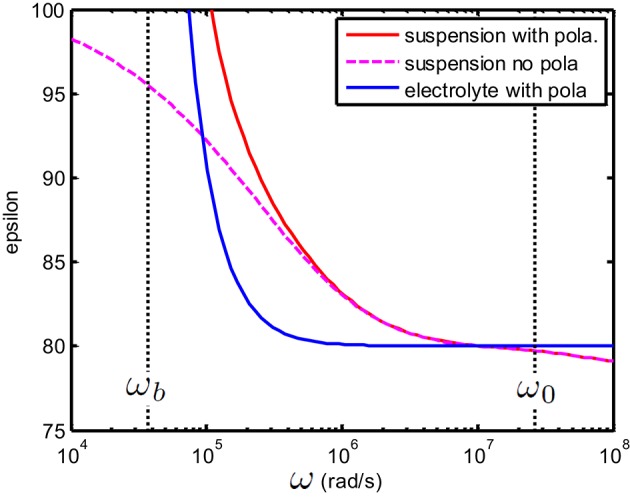
**Enlargement of Figure 5**. Above ω_*b*_ one finds that ε_*c,s*_ ≃ ε_*s*_ i.e., that the EP plays no role anymore. Note that for any frequency above ω_*P*_ the relation *K*_*c,s*_ ≃ *K*_*s*_ holds (see Figure 5). This implies that for frequencies above ω_*b*_ one has ${\tilde{K}}_{c,s}≃{\tilde{K}}_{s}$ (or equivalently ${\tilde{\varepsilon }}_{c,s}≃{\tilde{\varepsilon }}_{s}$). Similarly, above ω_*b*_ one has ${\tilde{K}}_{c,e}≃{\tilde{K}}_{e}$ which implies in particular for the present figure that ε_*c,s*_ ≃ ε_*e*_.

Equivalently,

$${\tilde{\varepsilon }}_{c,s}\left(\omega \right)≃{\tilde{\varepsilon }}_{s}\left(\omega \right)={\tilde{\varepsilon }}_{e}\left(\omega \right)\left(1+3\phi \tilde{\beta }\left(\omega \right)\right)$$

(40) $${\tilde{K}}_{c,s}\left(\omega \right)≃{\tilde{K}}_{s}\left(\omega \right)={\tilde{K}}_{e}\left(\omega \right)\left(1+3\phi \tilde{\beta }\left(\omega \right)\right)$$

from which $\tilde{\beta }$ can be directly deduced. This is illustrated in Figure 6 which is an enlargement of Figure 5.

The characteristic frequency ω_*b*_, representing the frequency above which EP becomes negligible, increases with ionic strength in the following way:

(41) $$\omega_{b}=\sqrt{\omega_{0}\omega_{P}}=\omega_{0}\sqrt{\frac{2}{\kappa d}}\sim \kappa^{3/2}$$

An indication for the values of ω_*b*_ is given in Table 2.

**Table 2:** **Values of ω_***b***_ as given by Equation (41), using a typical diffusion coefficient of $D_{0}=2.10^{-9}$ m^**2**^/s at room temperature, for 3 salt concentrations**.

***C_s_* (mM)**	**0.1**	**1**	**10**
ω_*b*_(*d* = 1 *mm*, rad/s)	1.6·10^4^	9.3·10^4^	5.2·10^5^
ω_*b*_(*d* = 10 *mm*, rad/s)	5.2·10^3^	2.9·10^4^	1.6·10^5^

Interestingly, one can verify that in most cases encountered in practice, one has ω_*b*_ ≲ (ω_*a*_, ω_0_). We will come back to this point in the following section.

**Figure 7:**
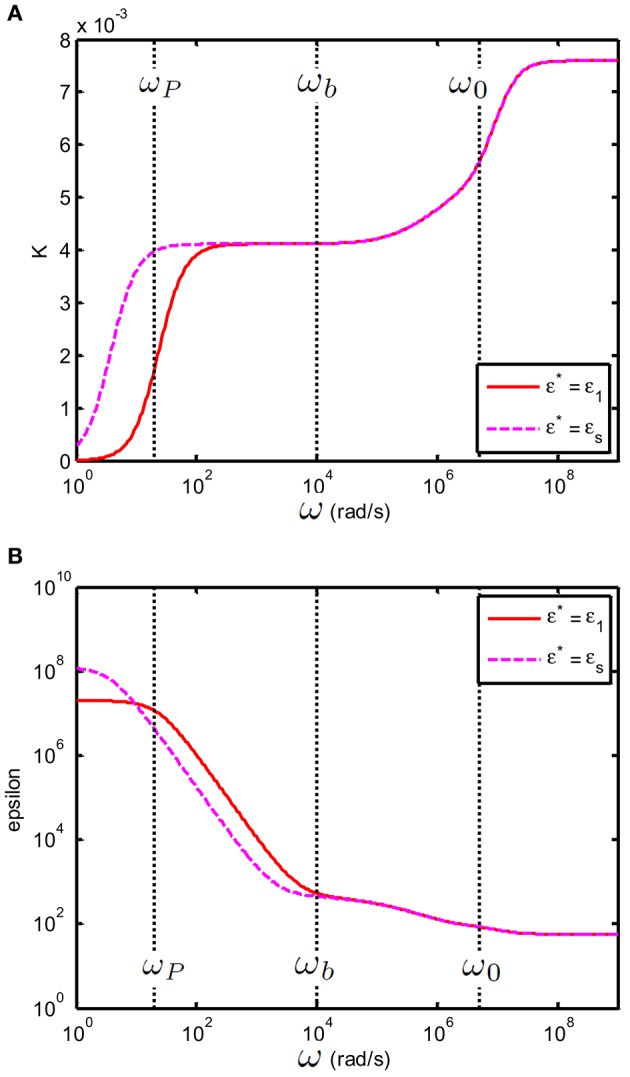
**(A)** Conductivity *K*_*c,s*_(S/m) and **(B)** relative permittivity (epsilon) ε_*c,s*_ of a suspension of 100 nm colloidal spheres (ϕ = 20%, *eζ*/*kT* = 4) in a 0.23 mM electrolyte solution of monovalent salt solution for which $D_{1}=2\times 10^{-9}$ m^2^/s and $D_{2}=1.98\times 10^{-9}$ m^2^/s. The spacing between electrodes is 10 mm. Red curve: the case where ε^*^ = ε_*e*_; Dashed magenta curve: the case where ε^*^ = ε_*s*_.

As above ω_*b*_ one has ${\tilde{K}}_{c,s}≃{\tilde{K}}_{s}$ this also implies that the value of ε^*^ only plays a role below ω_*b*_. This is illustrated in Figure 7. In order to better distinguish the features of the curves, we have chosen a large volume fraction (20%). This is permitted for the theoretical considerations presented, as using Equation (2) one can verify that |$\left|\phi \tilde{\beta }\right|<1$.

## 5. Accounting for electrode polarization

In this section, first three methods used to compensate experimentally for electrode polarization will be analyzed. In the last section, a general method to extract $\tilde{\beta }$ directly from the data will be presented. The frequency range of validity of each method is discussed.

### 5.1. Subtraction method

This method relies on the fact that EP can be modeled by a capacitance *C*_*EP*_ (see Section 3) associated with a relative permittivity $\varepsilon^{*}=\varepsilon_{e}$. The aim is to find a reference electrolyte solution (symbolized by the subscript *r*) that will give as best as possible the same *C*_*EP*_ as the one of the investigated suspension. From the section about equivalent circuits and from general definitions [see Equation (12) and Supplementary Material 1], we can write that (for *x* = *r* or *x* = *s*):

$${\tilde{Z}}_{c,x}\left(\omega \right)={\tilde{Z}}_{x}\left(\omega \right)+\frac{1}{i\omega C_{EP,x}}$$

(42) $$\frac{1}{{\tilde{Z}}_{x}\left(\omega \right)}=C{\tilde{K}}_{x}\left(\omega \right)$$

For the sake of argument, we will consider planar electrodes and use *C* = *S*/*d*. We then have:

$$C_{EP,s}=C\varepsilon_{0}\varepsilon_{e}\frac{\kappa_{s}d}{2}$$

(43) $$C_{EP,r}=C\varepsilon_{0}\varepsilon_{e}\frac{\kappa_{r}d}{2}$$

The ionic strength is determined by the amount of charge carriers in the system, which implies that conductivity and ionic strength are linked, see Equation (28) for electrolyte solutions for example. For the reference electrolyte, it is therefore found that $K_{r}\propto \kappa_{r}^{2}$. On the other hand, the conductivity for colloidal suspensions is given by Equation (8) i.e., at low frequencies:

(44) $$K_{s}\left(\omega \to 0\right)=K_{e}\left(1+3\phi \text{Re }\left[\tilde{\beta }\left(\omega \to 0\right)\right]\right)$$

Equation (44) gives the relation for the conductivity of the suspension after it has been mixed with the suspending electrolyte. The conductivity $K_{e}=\varepsilon_{0}\varepsilon_{e}\kappa_{s}^{2}D_{0}$ therefore represents the conductivity of the suspending electrolyte *including* all other ions (counterions and impurities) originating from the original (concentrated) suspension used to be mixed with the suspending electrolyte. In some cases, one can assume that the original suspension is devoid of impurities, and that the amount of counterions is negligeable compared to the amount of ions stemming from the electrolyte suspension. This last condition is usually true when dilute suspensions of large (> 10 nm) colloidal particles are studied. One can then safely assume that κ_*s*_ = κ_*e*_ i.e., the ionic strength of the suspension (symbolized by the subscript *s*) is given by the ionic strength of the suspending electrolyte (symbolized by the subscript *e*). In the case of nanocolloids, i.e., when there is a relative large total interface between particles and water and hence a non-negligible amount of counterions, or in the case that the original suspension of colloids (before it is added to the suspending electrolyte) contains impurities, i.e., ions that are not counterions, one can have κ_*s*_ ≠ κ_*e*_.

In practice, the value of κ_*s*_ can be difficult to obtain, because measuring the conductivity of a suspension does not give direct access to κ_*s*_. A procedure, based on experimental results, has consequently been developed to get a reference electrolyte solution for which in close approximation *C*_*EP,s*_ ≃ *C*_*EP,r*_ without the need of κ_*s*_ being known. This procedure is now outlined.

In the frequency range of interest, i.e., for ω_0_ ≫ ω ≫ ω_*P*_ we get from Equations (32), (35), and (37) for the suspension and the reference solution:

(45) $$\begin{array}{l} \;K_{c,x}≃K_{x}\;(x=s\text{ or }r)\\ \varepsilon_{c,r}\left(\omega \right)≃\frac{{\left(K_{r}\right)}^{2}}{{\left(\omega \varepsilon_{0}\right)}^{2}\varepsilon_{e}\kappa_{r}d/2}+\varepsilon_{e}\\ \varepsilon_{c,s}\left(\omega \right)≃\frac{{\left(K_{s}\left(\omega \right)\right)}^{2}}{{\left(\omega \varepsilon_{0}\right)}^{2}\varepsilon_{e}\kappa_{s}d/2}+\varepsilon_{s}\left(\omega \right) \end{array}$$

As discussed in the previous section, the conductivity in this frequency range is not affected by EP, only the permittivity is, in the range [ω_*P*_, ω_*b*_]. Above ω_*b*_ one has ε_*c,s*_(ω) ≃ ε_*s*_(ω). The subtraction method is therefore only applied to the permittivities, and should compensate for EP in the range [ω_*P*_, ω_*b*_]. The reference electrolyte solution is made of an electrolyte of the same type as the electrolyte present in the suspension (ex: if NaCl is the suspending electrolyte, NaCl will be taken as reference electrolyte). The concentration of the reference electrolyte is chosen such that the conductivity of the reference electrolyte *K*_*r*_ equals:

(46) $$K_{r}=K_{s}\left(\omega_{P}<\omega <\omega_{b}\right)$$

which implies that:

(47) $${\tilde{K}}_{r}\left(\omega \right)=K_{s}\left(\omega_{P}<\omega <\omega_{b}\right)+i\omega \varepsilon_{e}\varepsilon_{0}$$

In the range ω_*P*_ < ω < ω_*b*_ the conductivity *K*_*s*_(ω_*P*_ < ω < ω_*b*_) is constant. For frequencies smaller than ω_*P*_ the conductivity *K*_*c,s*_ is going to zero, as the electrodes are blocking, but above ω_*P*_ one has *K*_*c,s*_(ω) = *K*_*s*_(ω), as was shown in Section 3. One can verify from Equation (44) that below ω_*b*_ one has *K*_*s*_(ω) = *K*_*s*_ (i.e., *K*_*s*_ does not depend on frequency) provided that $\text{Re}\left(\tilde{\beta }\right)$ remains constant.$\;\text{Re}\left(\tilde{\beta }\right)$ is indeed a constant at low frequencies, and will start to exhibit relaxation phenomena when the lowest characteristic frequency associated with the colloidal particle is reached. This frequency is given by

(48) $$\omega_{a}=D_{0}/a^{2}$$

where *a* is the radius of the colloidal particle and *D*_0_ is given in Equation (14). This relaxation frequency occurs in the frequency regime [10^4^–10^6^] rad/s for particle sizes between [25–250] nm. In most cases encountered in experiments one has therefore ω_*a*_ > ω_*b*_ and $\text{Re}\left(\tilde{\beta }\right)$ is constant below ω_*b*_ implying that *K*_*s*_ is constant. For extreme cases, like very large particles at low ionic strength, the situation ω_*a*_ ≃ ω_*b*_ could occur in which case an option can be to increase *d* so as to lower ω_*P*_ and take *K*_*r*_ = *K*_*s*_(ω ≳ ω_*P*_).

As ϕ is supposed to be small, one has in the range ω_*P*_ < ω < ω_*b*_, in good approximation, $K_{s}≃K_{e}=\varepsilon_{0}\varepsilon_{e}\kappa_{s}^{2}D_{0}$ which implies that κ_*s*_ ≃ κ_*r*_. One can now get rid of the EP contribution in the whole frequency range ω_0_ ≫ ω ≫ ω_*P*_. By substracting ε_*c, r*_ from ε_*c,s*_ one obtains for ε_*s*_(ω):

(49) $$\varepsilon_{s}\left(\omega \right)=\varepsilon_{c,s}\left(\omega \right)-\varepsilon_{c,r}\left(\omega \right)+\varepsilon_{e}$$

**Figure 8:**
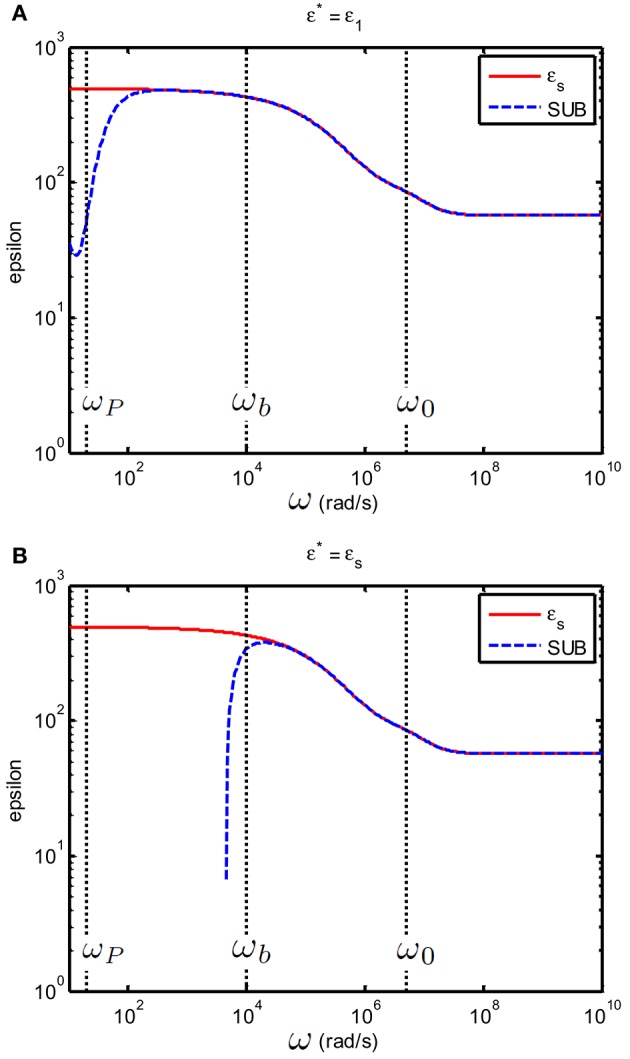
**Relative permittivity (epsilon) of a suspension of 100 nm colloidal spheres (ϕ = 20%, ***eζ***/***kT*** = 4) in a 0.23 mM electrolyte solution of monovalent salt solution for which $D_{1}=2\times 10^{-9}$ m^2^/s and $D_{2}=1.98\times 10^{-9}$ m^**2**^/s**. The spacing between electrodes is 10 mm. **(A)** The case where ε^*^ = ε_*e*_. **(B)** The case where ε^*^ = ε_*s*_ Red curves: ε_*s*_Blue curves: ε_*s*_ found by using the subtraction procedure

As stated at the beginning of this section, the subtraction method will not work when $\varepsilon^{*}\neq \varepsilon_{e}$ as this would imply that *C*_*EP,s*_ ≠ *C*_*EP,e*_. If one assumes that $\varepsilon^{*}=\varepsilon_{s}$ even though ε_*s*_ and ε_*e*_ differ by less than 10% this will give huge errors in the subtraction method, as Figure 8 illustrates. Note that around ω_*p*_ the values for the permittivity are of the order of 10^7^ (as can be seen in Figure 5). Substracting such huge numbers goes at the cost of accuracy: this explains the deviations observed the theoretical prediction (red line) and substraction method result (dashed blue line) at low frequencies.

As discussed after Equation (45), two main frequency regions can be distinguished in the spectrum. In the range [ω_*P*_, ω_*b*_] electrode polarization is dominant and we obtain from Equation (45):

(50) $$\varepsilon_{c,x}\left(\omega \right)≃\frac{{\left(K_{x}\left(\omega \right)\right)}^{2}}{{\left(\omega \varepsilon_{0}\right)}^{2}\varepsilon_{x}\kappa_{x}d/2}$$

where $\varepsilon_{x}=\varepsilon^{*}$ for suspensions and ε_*x*_ = ε_*e*_ for electrolytes. One might wonder whether it is possible to compensate for electrode polarization by dividing the dielectric spectrum of the suspension with the one of the solvent. This gives in the range [ω_*P*_, ω_*b*_]:

(51) $$\frac{\varepsilon_{c,s}}{\varepsilon_{c,e}}≃\frac{\varepsilon_{e}\kappa_{e}{\left(K_{s}\right)}^{2}}{\varepsilon^{*}\kappa_{s}{\left(K_{e}\right)}^{2}}$$

This ratio will probably be close to one, considering the discussion given above about the values of these variables. Clearly, this way of elimination of the electrode polarization contribution does not give access to ε_*s*_ in the frequency range where electrode polarization is prevailing. Above ω_*b*_ we have shown that electrode polarization does not play a significant role, and therefore we obtain from Equation (45):

(52) $$\frac{\varepsilon_{c,s}}{\varepsilon_{c,e}}≃\frac{\varepsilon_{s}}{\varepsilon_{e}}$$

Combining real and imaginary terms we obtain from Equations (12, 45) for the complex ratio, above ω_*b*_:

(53) $$\frac{{\tilde{\varepsilon }}_{c,s}}{{\tilde{\varepsilon }}_{c,e}}=\frac{{\tilde{K}}_{c,s}}{{\tilde{K}}_{c,e}}≃\frac{{\tilde{\varepsilon }}_{s}}{{\tilde{\varepsilon }}_{e}}=\frac{{\tilde{K}}_{s}}{{\tilde{K}}_{e}}$$

From Equations (2) and (5) one can see that above ω_*b*_ the ratios $\text{Re}\left({\tilde{K}}_{c,s}/{\tilde{K}}_{c,e}\right),$ or alternatively $\text{Re}\left({\tilde{\varepsilon }}_{c,s}/{\tilde{\varepsilon }}_{c,e}\right),$ provide direct information about $\text{Re}\left(\tilde{\beta }\right)$ whereas $\text{Im}\left({\tilde{K}}_{c,s}/{\tilde{K}}_{c,e}\right),$ or $\text{Im}\left({\tilde{\varepsilon }}_{c,s}/{\tilde{\varepsilon }}_{c,e}\right),$ provide direct information about $\text{Im}\left(\tilde{\beta }\right).$ By contrast, the ratios $\text{Re}\left({\tilde{\varepsilon }}_{c,s}\right)/\text{Re}\left({\tilde{\varepsilon }}_{c,e}\right)$ and $\text{Im}\left({\tilde{\varepsilon }}_{c,s}\right)/\text{Im}\left({\tilde{\varepsilon }}_{c,e}\right)$ are less appealing because they each depend on a mix of $\text{Re}\left(\tilde{\beta }\right)$ and $\text{Im}\left(\tilde{\beta }\right)$.

### 5.2. Variable electrode separation method

This technique is, in principle, extremely well-suited for compensating for electrode polarization. We start again from the general definitions:

$${\tilde{Z}}_{c,s}\left(\omega \right)={\tilde{Z}}_{s}\left(\omega \right)+\frac{1}{i\omega C_{EP,s}}$$

(54) $$\frac{1}{{\tilde{Z}}_{s}\left(\omega \right)}=C{\tilde{K}}_{s}\left(\omega \right)$$

For the sake of argument, we again consider planar electrodes and hence *C* = *S*/*d*. Moreover:

$$C_{EP,s}=C\varepsilon_{0}\varepsilon^{*}\frac{\kappa_{s}d}{2}$$

Combining these equations leads to:

(55) $$\frac{1}{{\tilde{K}}_{c,s}\left(\omega \right)}=\frac{1}{{\tilde{K}}_{s}\left(\omega \right)}+\frac{2}{i\omega \varepsilon_{0}\varepsilon^{*}\kappa_{s}d}$$

From the experimental data one can then estimate ${\tilde{K}}_{s}^{-1}$ (and hence ${\tilde{K}}_{s}$) from a linear regression of ${\tilde{K}}_{c,s}^{-1}$ as function of 1/*d*. This method is applicable for the whole range of frequency, however it requires the combination of both the real and imaginary part of ${\tilde{K}}_{c,s}$ in the analysis. In the frequency range of interest, i.e., for ω_0_ ≫ ω ≫ ω_*P*_, the variable electrode separation technique can be applied to the imaginary part of ${\tilde{K}}_{c,s}$ (which is related to ε_*c,s*_) only. Indeed, we have found that in the range of interest [see Equation (45)]:

$$K_{c,s}≃K_{s}$$

(56) $$\varepsilon_{c,s}\left(\omega \right)≃\frac{{\left(K_{s}\right)}^{2}}{{\left(\omega \varepsilon_{0}\right)}^{2}\varepsilon^{*}\kappa_{e}d/2}+\varepsilon_{s}\left(\omega \right)$$

It is therefore possible to “clean” the signal from electrode polarization by a linear regression of ε_*c,s*_ as function of 1/*d*, from which ε_*s*_ is then easily obtained. Although 2 electrode separations suffice in theory, in practice, 3 or 4 electrode separations are usually used to minimize the experimental error. The technique was introduced by Fricke and Curtis (1937), and used, for suspensions, in combination with the subtraction method (described in the previous section) by the Dutch groups (van der Touw and Mandel, 1971; van der Touw et al., 1975; Cirkel et al., 1997; Chassagne et al., 2002, 2003). This technique is also used by the groups in Princeton and Granada (Hollingsworth and Saville, 2003; Jimenez et al., 2007). For more recent work on the topic, and experimental limitations of the techniques, we refer to Hollingsworth and Saville (2004), Beltramo and Furst (2012), and Beltramo and Furst (2012).

### 5.3. Four electrode cell method

**Figure 9:**
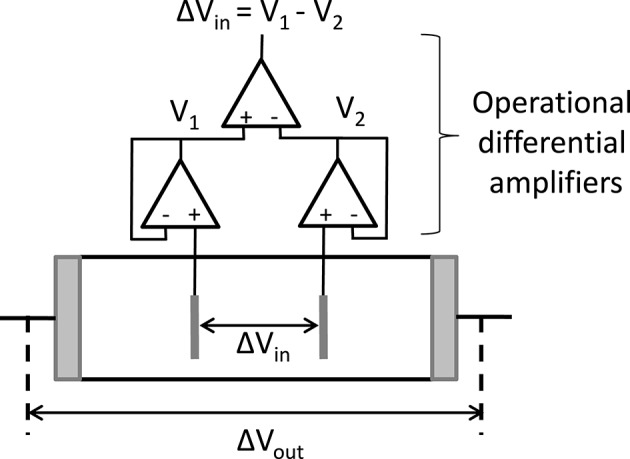
**Schematic representation of a 4-electrode cell; the voltage difference at the outer electrodes, where EP takes place, is ${\tilde{V}}_{o}=\Delta {\tilde{V}}_{out}={\tilde{Z}}_{o}\tilde{I}$ whereas the voltage difference at the inner (probing) electrodes is ${\tilde{V}}_{i}=\Delta {\tilde{V}}_{in}$; as EP should be minimized at the inner electrodes, the voltage difference measured at the inner electrodes ${\tilde{V}}_{i}$ should be done with virtually zero inner current (${\tilde{I}}_{i}≃0$), implying that the impedance ${\tilde{Z}}_{i}$ of the inner electrodes should be virtually infinite**. One then obtains, from the measurement of ${\tilde{V}}_{i}$ and $\tilde{I}$ : ${\tilde{Z}}_{s}={\tilde{V}}_{i}/\tilde{I}.$

The idea of using 4 electrodes stems from Schwan (1992). Four electrode cells are designed such that the two inner electrodes are able to measure a voltage difference that is devoid of EP. The outer electrodes are the current carrying electrodes, where EP occurs and causes an extra voltage drop close to these electrodes. Figure 9 sketches the measuring cell with 4 electrodes and equipped with special electronics. For the voltage sensing often needle-like electrodes are used so as to minimize their influence on the ionic flow. The inner electrodes should be designed such that the electric current generated by the outer electrodes is not disturbed. This requires that the voltage difference measured at the inner electrodes ${\tilde{V}}_{i}$ should be at virtually zero current (${\tilde{I}}_{i}≃0$), so that no current leaks away in the probing circuit. This implies that the impedance ${\tilde{Z}}_{i}$ of the inner electrodes should be virtually infinite (since ${\tilde{V}}_{i}={\tilde{Z}}_{i}{\tilde{I}}_{i}$). The measurement of ${\tilde{V}}_{i}$ can be achieved with the use of operational amplifiers. The current $\tilde{I}$ that is flowing in the whole cell can be measured with commercial frequency response analyzers or with impedance analyzers. We have:

(57) $$\begin{array}{l} {\tilde{V}}_{o}\left(\omega \right)={\tilde{Z}}_{c}\left(\omega \right)\tilde{I}\left(\omega \right)\\ {\tilde{V}}_{i}\left(\omega \right)={\tilde{Z}}_{ci}\left(\omega \right)\tilde{I}\left(\omega \right) \end{array}$$

where ${\tilde{Z}}_{c}$ is the impedance of the cell (with electrode polarization) and ${\tilde{Z}}_{ci}$ the impedance of the fluid between the inner electrodes. Since EP should not play a role at the inner measuring electrodes, we have ${\tilde{Z}}_{ci}={\tilde{Z}}_{s}$ i.e., the measured impedance ${\tilde{Z}}_{ci}$ gives directly the sought conductivity (or permittivity) of the suspension:

(58) $${\tilde{K}}_{s}\left(\omega \right)=C/{\tilde{Z}}_{ci}\left(\omega \right)={\tilde{K}}_{e}\left(\omega \right)(1+3\phi \tilde{\beta }\left(\omega \right))$$

Note that the cell constant *C* can be found separately from the impedance of an electrolyte with known electrical properties as the correct geometry of the probing electrodes might be unknown. We then simply have $C={\tilde{K}}_{e}\times {\tilde{Z}}_{ci}$, where ${\tilde{K}}_{e}=K_{e}+i\omega \varepsilon_{0}\varepsilon_{e}$ is known, and ${\tilde{Z}}_{ci}$ is measured.

Clearly, both the cell design and the electronic are more sophisticated than those of the common 2 electrode cell. Further details are given in Hayakawa et al. (1975) and Pelc et al. (2011). In the early publications gain/phase detectors were not available, so they were home built. Also, high technical skills in order to properly compensate for parasitic stray capacitances and inductions were required (Hayakawa et al., 1975; van der Touw et al., 1975). A new technique van der Ploeg and Mandel (1991) was proposed in 1991 to reduce the measurement time, and hence the possible drifts in conductivity. This technique is no longer in use as current set-ups are improved in such a way that the measurement time is not anymore an issue. Nowadays setting up 4 electrode experiments has become easier (Pelc et al., 2011). Four electrode cells are now also commercially available from e.g., Novocontrol.

It was already Schwan (1992) who warned that in practice ${\tilde{Z}}_{ci}$ could still contain some contribution of EP. This arises from the parasitic capacitance between the 4 electrodes. This extra parasitic EP can be compensated for by using the subtraction method explained above. This has been done by authors like Mandel, Saville, Hayakawa, and Kijlstra (Hayakawa et al., 1975; van der Touw et al., 1975; Myers and Saville, 1989; Kijlstra and Wegh, 1994). Critical remarks on this issue have also been made recently by Grimnes et al., Mazzeo et al., and Lvovich (Mazzeo, 2009; Lvovich, 2012; Grimnes and Martinsen, 2015). Another concern is that the 4 electrode system and its additional electronics is less suitable for high frequencies.

### 5.4. Logarithmic derivative method

This method was introduced by Jimenez et al. (2002, 2007) in order to compensate for EP, following the work of van Turnhout et al. (Wübbenhorst and van Turnhout, 2002). The logarithmic derivative method was originally derived to study the dielectric relaxation in polymers, but Jimenez et al. showed that it can be applied to colloidal suspensions as well, see also Kaatze et al. (Kaatze and Feldman, 2006).

These authors use the following definitions for the complex permittivity/conductivity of the system, which are different from Equations (6) :

(59) $$\begin{array}{l} \tilde{\varepsilon }\left(\omega \right)\equiv \varepsilon \prime \left(\omega \right)-i\varepsilon \prime \prime \left(\omega \right)\\ \tilde{K}\left(\omega \right)=K_{DC}+i\omega \varepsilon_{0}\tilde{\varepsilon }\left(\omega \right) \end{array}$$

implying in particular that:

(60) $${\tilde{K}}_{c}\left(\omega \right)=\left(K_{DC}+\omega \varepsilon_{0}\varepsilon_{c}^{\prime \prime }\left(\omega \right)\right)+i\omega \varepsilon_{0}\varepsilon_{c}^{\prime }\left(\omega \right)$$

As discussed earlier in the present article (see Section 2), the conductivity ${\tilde{K}}_{c}$ is zero at ω = 0 in the case of blocking electrodes; this implies that in our case *K*_*DC*_ = 0. We therefore obtain the equivalence, using Equations (6), (12), and (60):

(61) $$\begin{array}{l} K_{c}^{\prime }\left(\omega \right)=\omega \varepsilon_{0}\varepsilon_{c}^{\prime \prime }\left(\omega \right)\\ K_{c}^{\prime \prime }\left(\omega \right)=\omega \varepsilon_{0}\varepsilon_{c}^{\prime }\left(\omega \right) \end{array}$$

The logarithmic method is based on the use of a new variable defined by Wübbenhorst and van Turnhout (2002):

(62) $$\varepsilon_{D}^{\prime \prime }=\frac{-\pi }{2}\frac{\partial \varepsilon \prime }{\partial \text{ ln }\omega }$$

where the subscript “*D*” refers to “derivative.” This new variable $\varepsilon_{D}^{\prime \prime }\left(\omega \right)$ enables to better distinguish the characteristics relaxation frequencies of the system.

Despite its notation, $\varepsilon_{D}^{\prime \prime }$ differs from ε″. For a reference electrolyte solution of conductivity *K*_*r*_ one has, in the frequency range of interest:

(63) $$\varepsilon_{c,r}^{\prime }=\frac{2\pi }{\kappa d}\varepsilon_{e}{\left(\frac{\kappa^{2}D_{0}}{\omega }\right)}^{2}=\frac{2\pi }{\kappa d}\varepsilon_{e}{\left(\frac{K_{r}}{\varepsilon_{0}\varepsilon_{e}\omega }\right)}^{2}=\frac{2\pi }{\kappa d}\frac{1}{\varepsilon_{e}}{\left(\varepsilon_{c,r}^{\prime \prime }\right)}^{2}$$

This implies that:

(64) $$\begin{array}{l} \varepsilon_{D,c,r}^{\prime \prime }=\frac{-\pi }{2}\omega \frac{\partial \varepsilon_{c,r}^{\prime }}{\partial \omega }\\ \;=\frac{2\pi^{2}}{\kappa d}\varepsilon_{e}{\left(\kappa^{2}D_{0}\right)}^{2}\omega^{-2}\\ \;=\pi \varepsilon_{c,r}^{\prime }\\ \;=\frac{2\pi^{2}}{\kappa d}\frac{1}{\varepsilon_{e}}{\left(\varepsilon_{c,r}^{\prime \prime }\right)}^{2} \end{array}$$

This result is in agreement with the discussion in Wübbenhorst and van Turnhout (2002) about the expected quadratic dependence of $\varepsilon_{D}^{\prime \prime }$ on ε″ for sharp Debye like loss peaks. This quadratic dependence implies that ∂ε′/∂ ln ω provides a higher spectral resolution than ε″. It makes this quantity better suited for the decomposition of overlapping relaxations.

Let us say a few words about the origin of the use of ∂ε′/∂ ln ω (Steeman and van Turnhout, 1994). It was ∂ε′/∂ ln ω that enabled the authors of Steeman and van Turnhout (1994) to detect in its very spectrum for solid polymers just beyond the main-chain or α-relaxation, the space charge or ρ-relaxation, which arises from the motion of ions at high temperature and low frequencies. By contrast, the ρ-relaxation could not be seen at all in the ε″-spectra. Its better revealing power is furthermore due to another special feature of ∂ε′/∂ ln ω, namely that it does not contain any contribution of ohmic conduction. The conduction loss does add up however in the ε″ data, in which it often overshadows the genuine relaxation losses at low frequencies.

If the relaxation of a system stems from a variety of processes, then we better model the total response with a logarithmic distribution of relaxation times. Both ∂ε′/∂ ln ω and ε″ can be linked to this distribution (Steeman and van Turnhout, 1994). In fact, they provide approximations to it, −∂ε′/∂ ln ω gives a first order and ε″ a zero order estimate. This explains once more that ∂ε′/∂ ln ω has a higher resolving power than ε″.

Both approximations become the better, the broader the distribution. For broad dielectric distributions we can therefore envisage −∂ε′/∂ ln ω as being a close approximation to π/2ε″. It was for this reason that the symbol $\varepsilon_{D}^{\prime \prime }$ was used for (−π/2)∂ε′/∂ ln ω.

**Figure 10:**
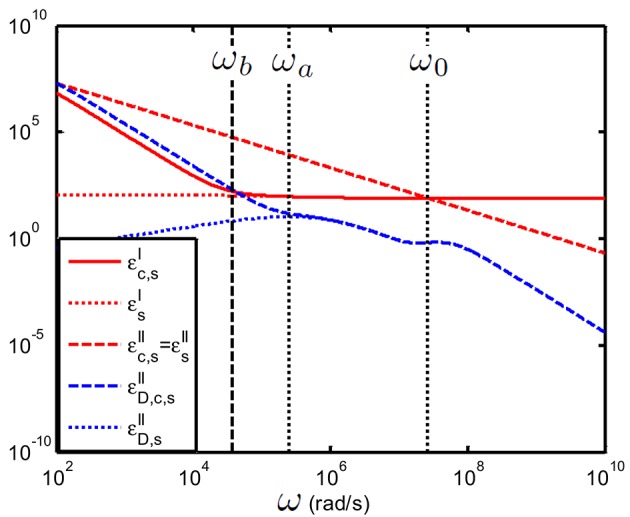
**Comparison between $\varepsilon_{D,s}^{\prime \prime }$, $\varepsilon_{D,c,s}^{\prime \prime }$, $\left(\varepsilon_{s}^{\prime },\varepsilon_{s}^{\prime \prime }\right)$ and $\left(\varepsilon_{c,s}^{\prime },\varepsilon_{c,s}^{\prime \prime }\right)$ as function of frequency; suspension of 100 nm colloidal spheres (ϕ = 1%, *eζ*/*kT* = 4) in a 1 mM electrolyte solution of monovalent salt solution for which $D_{1}=2\times 10^{-9}$ m^2^/s and $D_{2}=1.98\times 10^{-9}$ m^2^/s**. The spacing between the electrodes is 10 mm. Using the logarithmic derivative method (in blue) enables to better distinguish the relaxation processes associated with the colloidal particles.

In Figure 10 we have considered a colloidal suspension. One can see that indeed $\left(\varepsilon_{D,c,s}^{\prime \prime },\varepsilon_{D,s}^{\prime \prime }\right)$ display clear relaxation phenomena, whereas these dispersions cannot be seen in the variables $\left(\varepsilon_{s}^{\prime },\varepsilon_{s}^{\prime \prime }\right)$ and $\left(\varepsilon_{c,s}^{\prime },\varepsilon_{c,s}^{\prime \prime }\right)$.

This high-resolution property of $\varepsilon_{D}^{\prime \prime }$ therefore enables us to identify the two important relaxations frequencies occurring in a colloidal suspension, namely $\omega_{a}=D_{0}/a^{2}$ and ω_0_ [see Equations (26) and (48)] where *a* is the radius of the colloidal particle and *D*_0_ is given in Equation (14). The relaxation frequency ω_*a*_ shows up in the frequency regime [10^4^–10^6^] rad/s for particle sizes between [25–250] nm. The second relaxation frequency, $\omega_{0}=D_{0}\kappa^{2},$ is associated with the colloidal particle double layer. The characteristic length scale related to this frequency is the Debye length κ^−1^. Because the electrolyte that determines the conductivity of the bulk electrolyte is the same as the one in which the colloidal particles are suspended, ω_0_ is a characteristic frequency both for the electrolyte solution (as in this case $\omega_{0}=K_{e}/\left(\varepsilon_{0}\varepsilon_{e}\right)=D_{0}\kappa^{2}$) and for the colloidal particles. We have seen in Table 1 that in general ω_0_ ≥10^6^ rad/s. Note that for nanoparticles the two characteristic frequencies might overlap: $\omega_{a}≃\omega_{0}≃10^{7}$ rad/s for 10 nm particles in an 1 mM salt. An important relaxation feature present in the spectrum is due to EP. EP is linked to ω_*b*_ as defined in the previous section, see Equation (38). We have already noted that in many experimental conditions ω_*b*_ ≲ (ω_*a*_, ω_0_). We will show that in the cases where this inequality applies, it is possible to make use of this fact by performing the fits on the measured data *uncorrected* for EP.

In the following Sections 5.4.1 and 5.4.2, we show how the logarithmic derivative method can be applied when the relation between ${\tilde{\varepsilon }}_{s}$ and $\tilde{\beta }$ is known (section The direct fitting method) and when it is not known (The general fitting method). This last section is based on the work of Jimenez et al. (2007).

The “experimental data” needed in these sections to perform the fits are generated numerically using an equivalent circuit model, with the elements given in Equation (37). The necessary parameters are given in the legends of the figures. The “experimental data” mimic the response $\left(\varepsilon_{c,s}^{\prime },\varepsilon_{c,s}^{\prime \prime }\right)$ of a colloidal suspension (including EP). From the $\varepsilon_{c,s}^{\prime }$ thus obtained, one can derive

(65) $$\varepsilon_{D,c,s}^{\prime \prime }=\frac{-\pi }{2}\frac{\partial \varepsilon_{c,s}^{\prime }}{\partial \text{ ln }\omega }$$

#### 5.4.1. The direct fitting method (with a dipolar coefficient model)

Theories have been developed to express $\tilde{\beta }$ in terms of the relevant parameters of the colloidal particles, namely particle size, zeta potential or surface charge and Stern layer parameters. These theories have been derived for the case of colloidal suspensions consisting of spherical, homogeneously charged particles (DeLacey and White, 1981; Delgado, 2002; Ohshima, 2006; Chassagne and Bedeaux, 2008). Some work has also been performed on homogeneously charged spheroids, see Chassagne and Bedeaux (2008) and references within. An analytical expression for $\tilde{\beta }$ in the case of spheres can be found in Supplementary Material 1. Other (numerical) models can be found in Mangelsdorf and White (1990) and Minor et al. (1998). We are going to use the analytical expression given in Supplementary Material 1, but we emphasize that any other theory can be applied, even numerical ones, even though the fitting procedure becomes more challenging in this case. Equation (8) is used to link ${\tilde{\varepsilon }}_{s}$ (${\tilde{K}}_{s}$) to $\tilde{\beta }$. The logarithmic derivative $\varepsilon_{D,s}^{\prime \prime }$ can be calculated numerically from $\varepsilon_{s}^{\prime }\left(\tilde{\beta }\right)$ using the relation

(66) $$\varepsilon_{D,s}^{\prime \prime }=\frac{-\pi }{2}\frac{\partial \varepsilon_{s}^{\prime }}{\partial \text{ ln }\omega }$$

The $\varepsilon_{D,s}^{\prime \prime }$ thus obtained is a function of $\tilde{\beta }$ and therefore a function of the relevant parameters of the colloidal particles, the zeta potential in particular.

**Figure 11:**
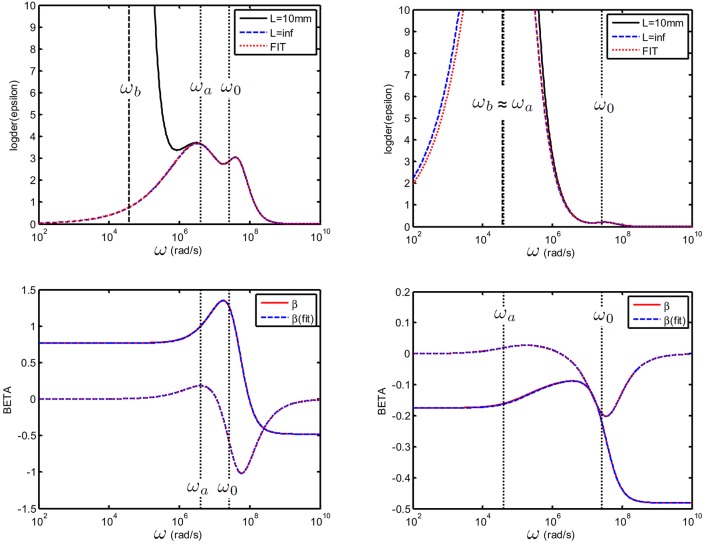
**Top:** Logarithmic derivative $\varepsilon_{D}^{\prime \prime }$ for a suspension consisting colloidal spheres (ϕ = 1%, *eζ*/*kT* = 4) in a 1 mM electrolyte solution of monovalent salt for which *D*_1_ = 2 × 10^−9^ m^2^/s and *D*_2_ = 3 × 10^−9^ m^2^/s. The electrode spacing is 10 mm. (left): 25 nm particles and (right): 250 nm particles. The fit with the dipolar coefficient was done between [ω_*b*_ = 2.5 × 10^*6*^-10^*10*^] rad/s using (ζ, *a*) as adjustable parameters. **Bottom**: Recalculated $\tilde{\beta }$ (blue) and original beta (red).

In Figure 11 we show two examples of the fit of $\varepsilon_{D,c,s}^{\prime \prime }$ (black curve) using the $\varepsilon_{D,s}^{\prime \prime }$ defined just above (red curve). The blue curve indicates the $\varepsilon_{D,s}^{\prime \prime }$ calculated using the equivalent circuit defined in Section 4.2 with *C*_*EP*_ = 0 which is equivalent to take an infinite electrode spacing.

For the two cases, which span a large particle size distribution at moderate ionic strength (1 mM), we are in the situation where ω_*a*_ ≳ ω_*b*_ (left figure) and ω_*a*_ ≃ ω_*b*_ (right figure). For all curves, the fits were always performed using the data in the frequency range above ω_*b*_. Different situations were tested: we checked that is is possible to fit the data using (a) the zeta potential ζ as single adjustable parameter, (b) ζ and the particle size *a* as adjustable parameters. Both gave the same excellent fit as in Figure 11. The fits were also possible adding small random errors to the data (not shown). We checked that it is possible to use 3 adjustable parameters: ζ, *a* and a Stern layer parameter *St*, defined in Supplementary Material 1, if a Stern layer conductance is introduced in $\tilde{\beta }$ and similarly introduced in the numerically generated “experimental data” (not shown).

Interestingly, when ω_*a*_ ≃ ω_*b*_ (see Figure 11), the direct fitting method gives very good results despite the fact that only a part of the first relaxation peak is fitted. This is because $\varepsilon_{s}^{\prime }\left(\tilde{\beta }\right)$ contains all possible relaxations processes of the colloid, and is therefore versatile enough to give a proper fit with few parameters.

#### 5.4.2. The general fitting method

This procedure, introduced by Jimenez et al. (2002, 2007) is based on two steps. The first one is to remove the electrode polarization (EP) contribution from the logarithmic derivative $\varepsilon_{D,c,s}^{\prime \prime }$ data in order to assess $\varepsilon_{D,s}^{\prime \prime }$, and the second step is to fit $\varepsilon_{D,s}^{\prime \prime }$ using a using a Havriliak-Negami (HN) relaxation function. This method is quite general, and can in theory be applied to a variety of complex fluids, such as polymeric or colloidal suspensions. The use of the Havriliak-Negami (HN) function originates from work done to describe empirically the dielectric relaxation of polymers (Havriliak and Negami, 1967). Jimenez et al. showed it is applicable to colloidal suspensions, and we refer to their work for more details and examples of application of the method (Jimenez et al., 2002, 2005, 2007).

In order to remove the EP contribution to $\varepsilon_{D,c,s}^{\prime \prime }$ Jimenez et al. introduce the relation:

(67) $$\varepsilon_{D,c,s}^{\prime \prime }\left(\omega \right)=A\omega^{-m}+\varepsilon_{D,s}^{\prime \prime }\left(\omega \right)$$

where *m* should be close to 2 [see Equation (64)], as *Aω*^−*m*^ represents the EP contribution (*A* is a parameter to be fitted to the data). The contribution *Aω*^−*m*^ is fitted to the data in the low-frequency part (assuming that $A\omega^{-m}\gg \varepsilon_{D,s}^{\prime \prime }$ in this part). The permittivity $\varepsilon_{D,s}^{\prime \prime }$, (named $\varepsilon_{D,cor}^{\prime \prime }$ by Jimenez et al.) represents the $\varepsilon_{D}^{\prime \prime }$ of the suspension in the absence of EP. It is obtained by subtraction over the whole frequency range ($\varepsilon_{D,s}^{\prime \prime }=\varepsilon_{D,c,s}^{\prime \prime }-A\omega^{-m}$).

It is clear that the logarithmic derivative method gives $\varepsilon_{s}^{\prime }=\varepsilon_{s}$ without its ω-independent part. The same holds for $\varepsilon_{HN,s}^{\prime }$ i.e., the $\varepsilon_{s}^{\prime }$ found from the logarithmic derivative of corresponding HN function. The expression for the HN function reads:

(68) $${\tilde{\varepsilon }}_{HN}\left(\omega \right)=\varepsilon_{HN}^{\prime }\left(\omega \right)-i\varepsilon_{HN}^{\prime \prime }\left(\omega \right)=\frac{\varepsilon_{HN}\left(0\right)-\varepsilon_{HN}\left(\infty \right)}{{\left[1+{\left(i\omega \tau \right)}^{a}\right]}^{b}}\;+\;\varepsilon_{HN}\left(\infty \right)$$

(Note that for *b* = 1 the function is called the Cole-Cole function while for *a* = *b* = 1 the function is called the Debye function). We can therefore define:

(69) $$\varepsilon_{D,HN,s}^{\prime \prime }\left(\omega \right)=\frac{-\pi }{2}\frac{\partial }{\partial \text{ ln }\omega }\left[\text{Re }\left(\frac{\varepsilon_{HN,s}\left(0\right)-\varepsilon_{HN,s}\left(\infty \right)}{{\left[1+{\left(i\omega \tau \right)}^{a}\right]}^{b}}\right)\right]$$

The parameters Δε_*HN, s*_ = ε_*HN, s*_(0) − ε_*HN, s*_(∞), τ, *a* and *b* are to be fitted to the data (ε_*HN, s*_(∞), being a constant, is not included in the fit). The missing constant is found from $\varepsilon_{HN,s}\left(\infty \right)=\varepsilon_{s}^{\prime }\left(\infty \right)$ because $\varepsilon_{s}^{\prime }$ reaches a constant at high frequencies. Indeed, we then have from Equation (2): $\varepsilon_{s}^{\prime }\left(\omega \to \infty \right)=\varepsilon_{e}\left(1+3\phi \text{Re}\left(\tilde{\beta }\left(\omega \to \infty \right)\right)\right)=\varepsilon_{\infty }^{\prime }$. From the parameters found from the fit of $\varepsilon_{D,s}^{\prime \prime }$ by $\varepsilon_{D,HN,s}^{\prime \prime }$ one then finds $\left[\varepsilon_{HN,s}^{\prime }-\varepsilon_{HN}\left(\infty \right)\right]=\left[\varepsilon_{s}^{\prime }-\varepsilon^{\prime }\left(\infty \right)\right]$ using Equation (68). From Equation (68), we also find that $\varepsilon_{HN,s}^{\prime \prime }\left(\omega \to 0\right)=0$. This implies that we can make the equivalence :

(70) $$\varepsilon_{HN,s}^{\prime \prime }\left(\omega \right)=\left(K_{s}\left(\omega \right)-K_{r}\right)/\left(\omega \varepsilon_{0}\right)$$

where *K*_*r*_ = *K*_*s*_(ω → 0). The electrolyte with a conductivity having this property has been termed reference electrolyte in the Section 5.1.

We define the increment $\Delta {\tilde{K}}_{HN,s}$ which can be found directly from the parameters of the fit using Equation (68):

(71) $$\begin{array}{l} \Delta {\tilde{K}}_{HN,s}\left(\omega \right)=\left[\varepsilon_{HN,s}^{\prime }\left(\omega \right)-\varepsilon_{HN,s}\left(\infty \right)-i\varepsilon_{HN,s}^{\prime \prime }\left(\omega \right)\right]\;(i\omega \varepsilon_{0})/\phi \\ \;=\left[\left(K_{s}\left(\omega \right)-K_{r}\right)+i\omega \varepsilon_{0}\left(\varepsilon_{s}\left(\omega \right)-\varepsilon_{s}\left(\infty \right)\right)\right]/\phi \\ \;=\left[{\tilde{K}}_{s}\left(\omega \right)-{\tilde{K}}_{r}\left(\omega \right)-i\omega \varepsilon_{e}\varepsilon_{0}3\phi \beta_{\text{inf}}\right]/\phi \end{array}$$

where ${\tilde{K}}_{r}\left(\omega \right)=K_{r}+i\omega \varepsilon_{0}\varepsilon_{e}$ and where we have used, for a colloidal sphere with relative permittivity ε_2_ [see Supplementary Material 1 for a general expression of $\tilde{\beta }\left(\omega \right)$]

(72) $$\begin{array}{l} \varepsilon_{s}\left(\infty \right)=\varepsilon_{e}\left(1+3\phi \beta_{\text{inf}}\right)\\ \;\beta_{\text{inf}}=\frac{\varepsilon_{2}-\varepsilon_{e}}{\varepsilon_{2}+2\varepsilon_{e}} \end{array}$$

From Equation (2) we deduce that (we recall that ${\tilde{K}}_{e}={\tilde{K}}_{1}$):

(73) $$\tilde{\beta }\left(\omega \right)=\frac{{\tilde{K}}_{s}\left(\omega \right)-{\tilde{K}}_{1}\left(\omega \right)}{3\phi {\tilde{K}}_{1}\left(\omega \right)}$$

Combining Equations (71) and (73), it is possible to find the required dipole coefficient $\tilde{\beta }$ by using the relation:

(74) $$\tilde{\beta }\left(\omega \right)=\frac{\phi \Delta {\tilde{K}}_{HN}\left(\omega \right)+{\tilde{K}}_{r}\left(\omega \right)+i\omega \varepsilon_{e}\varepsilon_{0}3\phi \beta_{\text{inf}}-{\tilde{K}}_{1}\left(\omega \right)}{3\phi {\tilde{K}}_{1}\left(\omega \right)}$$

Note that ${\tilde{K}}_{r}$ and ${\tilde{K}}_{1}$ can be obtained by measuring the conductivities of the corresponding electrolytes. In order to estimate β_inf_ only the value of ε_2_ is required (the value of the relative permittivity of water, i.e., ε_*e*_, is known). It is also possible to determine β_inf_ experimentally from

(75) $$\beta_{\text{inf}}=\text{Re }\left(\tilde{\beta }\left(\omega \to \infty \right)\right)=\frac{\varepsilon_{s}^{\prime }\left(\omega \to \infty \right)-\varepsilon_{e}}{3\phi \varepsilon_{e}}$$

**Figure 12:**
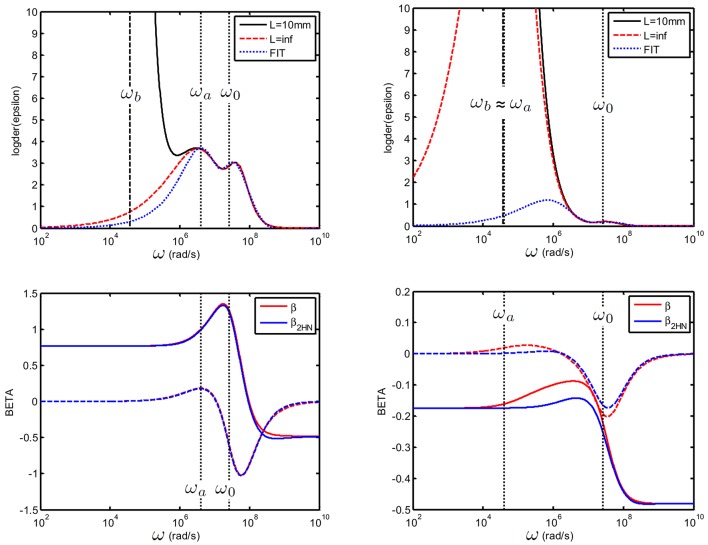
**Top:** Logarithmic derivative $\varepsilon_{D}^{\prime \prime }$ for a suspension consisting of colloidal spheres(ϕ = 1%, *eζ*/*kT* = 4) in a 1 mM electrolyte solution of monovalent salt for which *D*_1_ = 2 × 10^−9^ m^2^/s and *D*_2_ = 3 × 10^−9^ m^2^/s. (left): 25 nm particles and (right): 250 nm particles. The electrode spacing is 10 mm, and the 2 HN fit was done between [2.5.10^*6*^–10^*10*^] rad/s. **Bottom**: recalculated $\tilde{\beta }$ [blue line, from Equation (74) and original beta (red)]: despite the inaccuracy in the 2 HN fit, the error in $\tilde{\beta }$ is small, see explanation in text.

As discussed at the beginning of this section, there are two characteristic frequencies associated with the colloidal particle's relaxations. It is therefore possible to fit both relaxation frequencies when they are experimentally accessible, by using a sum of two HN functions such that:

(76) $${\tilde{\varepsilon }}_{HN}=\frac{\Delta \varepsilon_{HN,1}}{{\left[1+{\left(i\omega \tau_{1}\right)}^{a_{1}}\right]}^{b_{1}}}+\frac{\Delta \varepsilon_{HN,2}}{{\left[1+{\left(i\omega \tau_{2}\right)}^{a_{2}}\right]}^{b_{2}}}+\varepsilon_{HN}\left(\infty \right)$$

which implies the use of 8 fitting parameters: Δε_*HN*, 1_, τ_1_, *a*_1_, *b*_1_, Δε_*HN*, 2_, τ_2_, *a*_2_, *b*_2_. Equations (71)–(74) can subsequently be applied to find $\tilde{\beta }$. We have checked that this procedure gives good results, see Figure 12). In this example, we have fitted the uncorrected measured data, in the frequency range above ω_*b*_. For the smallest particle used (25 nm) ω_*a*_ ≳ ω_*b*_ the fit is very good. For larger colloidal particles (250 nm particles), we find that despite the fact that ω_*a*_ ≃ ω_*b*_ and that the double HN is less versatile than the dipolar coefficient used in the direct fitting method discussed in the section above, the result of the fit is surprisingly good, especially at low frequencies. This is due to the fact that the value of $\tilde{\beta }$ at low frequencies depends nearly exclusively on the value of *K*_*r*_ = *K*_*s*_(ω → 0) which is not affected by EP, and does not depend on $\Delta {\tilde{K}}_{HN}$:

(77) $$\begin{array}{l} \text{Re }\left(\beta \left(\omega \to 0\right)\right)=\frac{K_{s}\left(\omega \to 0\right)-K_{e}}{3K_{e}\phi }\\ \text{Im }\left(\beta \left(\omega \to 0\right)\right)=0 \end{array}$$

The correct fit of the second peak (associated with the double layer relaxation and ω_0_) is, on the other hand, very important to get the correct relaxation of $\tilde{\beta }$ in the high frequency range.

## 6. Conclusions

In this article, we have discussed the dielectric spectroscopy response of electrolytes and colloidal suspensions. We concentrated ourselves on the elimination of the phenomenon called “electrode polarization” (EP) that can overshadow the measured signal. This phenomenon is caused by the build-up of charges close to the electrodes in the suspension (or electrolyte solution). We have not discussed the other source of unwanted effects, namely stray impedances that originate from the non-ideality of the experimental set-up, and that also affect the measured signal. From the data, important parameters for the characterization of colloidal particles can be assessed. These parameters are found from the evaluation of the dipolar coefficient $\tilde{\beta }$ which is obtained from the cleaned and/or fitted data.

It was found that several characteristic frequencies could be identified and linked to EP effects. An important frequency is $\omega_{b}=\sqrt{\omega_{0}\omega_{P}}$ with ω_*P*_ = 2κ*D*_±_/*d* and $\omega_{0}=\kappa^{2}D_{\pm }$ where κ is the inverse of the Debye length, *D*_±_ the characteristic ionic diffusion coefficient and *d* the distance between electrodes (planar electrodes) or the smallest of the two radii in the case of cylindrical electrodes. Above ω_*b*_ the measured data are not affected anymore by EP. This enables, for many cases encountered in practice, to assess the properties of the colloidal particles by analyzing directly the data uncorrected for EP. This is shown in the last section. In that section, two fitting procedures are described: first, we consider “the direct fitting method” that makes use of existing dipolar coefficient models for colloidal suspensions, and then we explain the “general fitting method” which is based on the logarithmic derivative method and makes use of Havriliak-Negami (HN) relaxation functions. This last method is especially suited when the fluid under investigation is complex, i.e., when no good model for its behavior as function of the frequency exists. The correction of the experimental data for EP with the logarithmic derivative thus is quite successful. It has the advantage that it can be achieved simply by invoking ∂ε′/∂ ln ω in the data analysis. No special adaptations have to be made to the measurement or the equipment.

Also some experimental techniques enable us to compensate for EP. In this article, we tested them from a theoretical perspective. As outlined in the corresponding sections, even though a model could be ideal to compensate for EP in a given range of frequency, there are often technical limitations that could limit their validity. However, from the theoretical modeling, we have found that:

1 - The “subtraction method” can compensate for electrode polarization above the critical frequency ω_*P*_ provided that the dielectric permittivity ε^*^ in the small slab of liquid adjacent to the electrodes is given by ε_*e*_. If this permittivity differs from ε_*e*_, the subtraction method is not suited for eliminating EP. The fact that ε^*^ may or may not be affected by the presence of colloidal particles in the slab of liquid adjacent to the electrodes is still an open question.

2 - The “variable electrode separation method” is well-suited for compensating for electrode polarization over the whole frequency range.

3 - The “4-electrode cell method” gives directly access to the wanted signal, devoid of EP over the whole frequency range.

## Author contributions

All the authors made substantial contributions to the conception of the work; They all helped revising it critically for important intellectual content; They all approved the final version to be published; They agree to be accountable for all aspects of the work in ensuring that questions related to the accuracy or integrity of any part of the work are appropriately investigated and resolved.

### Conflict of interest statement

The authors declare that the research was conducted in the absence of any commercial or financial relationships that could be construed as a potential conflict of interest.
