# Supplementary material for: Compensating for Electrode Polarization in Dielectric Spectroscopy Studies of Colloidal Suspensions: Theoretical Assessment of Existing Methods
Source: Front Chem. 2016 Jul 19;4:30. doi: 10.3389/fchem.2016.00030 (PMC4949231; doi:10.3389/fchem.2016.00030)
Supplement: Supplementary file 1 [file DataSheet1.pdf]

Supplementary material 1: Explicit model for the dipolar coefficient

The dipolar coefficient, which is a complex function, is an important quantity because it rules the relaxation behavior of a suspension. It depends not only on the properties of the colloidal particles, but also on their (interfacial) interaction with the electrolyte. It depends on important parameters like the zeta potential , which is a measure for the charge on the particles, Stern layer parameters and the particle radius a. The general expression for the complex conductivity of a suspension of colloidal spheres of volume fraction ϕ is:

$$\widetilde{K}(\omega) = \widetilde{K}_e(\omega) \frac{1 + 2\phi\widetilde{\beta}(\omega)}{1 - \phi\widetilde{\beta}(\omega)} \tag{78}$$

which for small ϕ reduces to:

$$\widetilde{K}(\omega) = \widetilde{K}_e(\omega) \left(1 + 3\phi\widetilde{\beta}(\omega)\right) \tag{79}$$

The associated complex permittivity reads:

$$\widetilde{\varepsilon}(\omega) = \frac{\widetilde{K}_e(\omega)}{i\omega\varepsilon_0} \left(1 + 3\phi\widetilde{\beta}(\omega)\right) \tag{80}$$

Both $\widetilde{K}$ and $\widetilde{\varepsilon}$ are no longer labelled with subscript "s" in order to keep the notation short. The complex conductivity of the bulk electrolyte $\widetilde{K}_e$ is given by:

$$\widetilde{K}_e(\omega) = K_e + i\varepsilon_0\varepsilon_e\omega \tag{81}$$

where K_e is given in eq.(29). The complex dipolar coefficient $\widetilde{\beta}$ is given by [17]:

$$\widetilde{\beta}(\omega) = \frac{\widetilde{K}_2 - \widetilde{K}_e + 2\left[\widetilde{K}_{//} + \widetilde{K}_U + \widetilde{K}_{//}^{ex}\right] + \left[\widetilde{K}_\perp + \widetilde{K}_\perp^{ex}\right]}{\widetilde{K}_2 + 2\widetilde{K}_e + 2\left[\widetilde{K}_{//}(a/r_0)^3 + \widetilde{K}_U(a/r_1)^3 + \widetilde{K}_{//}^{ex} - \widetilde{K}_\perp - \widetilde{K}_\perp^{ex}\right]} \tag{82}$$

The complex conductivity from the core of the insulating (dielectric) colloidal particle is given by:

$$\widetilde{K}_2(\omega) = i\varepsilon_0\varepsilon_2\omega \tag{83}$$

The other conductivities which depend on the zeta potential ζ, the particle radius a and the inverse of the Debye length κ^{-1}, are defined by:

$$\begin{aligned}
\widetilde{K}_{//}(\omega) &= -K_e I_{n,eq} - \frac{2J_1\left[I_{c,eq}^2 - I_{n,eq}^2\right]}{J_2(r_0/a)^3 \exp(\lambda_n(r_0 - a))} \\
\widetilde{K}_\perp(\omega) &= \frac{2J_1 K_e I_{n,eq}}{J_2(r_0/a)^3 \exp(\lambda_n(r_0 - a))} \\
\widetilde{K}_U(\omega) &= -K_e m \frac{e\zeta}{kT} I_{c,eq}\left[\frac{J_1}{2J_2(r_0/a)^3 \exp(\lambda_n(r_0 - a))} - 1\right]
\end{aligned} \tag{84}$$

where λ_n is defined by eq.(17). The potential ζ is defined as the absolute value of the zeta potential. Note that in $\widetilde{K}_U$ of [17] (eq.(67) in that article) the negative sign is missing. We stress that except for this minus sign, the expression for $\widetilde{K}_U$ is correct. Rica et al. [47] suggested another correction. This originates from an incorrect approximation for the electrophoretic mobility flow given in [17]. We found that using the expressions given above, one gets the same dipolar coefficient as the one found by solving numerically the set of electrokinetic equations, as done by DeLacey and White [16]. The complex conductivities labelled "ex" (as "extra" conductivities) can be used to represent Stern layer conductivities. When no

Stern layer is present these conductivities are taken equal to zero. When Stern layer conductivities are used, they are often expressed as real and frequency-independent values and can be given by [13],[17]:

$$\begin{aligned}\widetilde{K}_{//}^{ex} &= K_{//}^{ex} = StK_e \\ \widetilde{K}_{\perp}^{ex} &= K_{\perp}^{ex} = K_{//}^{ex}\end{aligned} \tag{85}$$

where St is an adjustable parameter. The Stern layers conductivities therefore only introduce a single unknown parameter. The other relevant parameters, discussed in more detail in [17], are:

$$\begin{aligned}J_1 &= 1 + \lambda_n r_0 \\ J_2 &= 1 + (1 + \lambda_n a)^2 \\ r_0 &= a + \left(1 + \frac{3}{\kappa a}\exp\left(\frac{-e\zeta}{2kT}\right)\right)\kappa^{-1} \\ r_1 &= a + \frac{2.5}{1 + 2\exp(-\kappa a)}\kappa^{-1} \\ m &= \frac{2}{3}\frac{\varepsilon_0\varepsilon_e (kT)^2}{\eta D_0 e^2}\end{aligned} \tag{86}$$

where κ is given by eq.(21). The variables $I_{c,eq}$ and $I_{n,eq}$ in eq.(84) are in fact integrals. They depend on ζ and κa and can be found from:

$$\begin{aligned}I_{c,eq} &= \frac{1}{(\kappa a)^2}\int_{\kappa a}^{\kappa r_0} x\sinh\left(\Psi_{eq}^*\right)dx \\ I_{n,eq} &= \frac{-1}{(\kappa a)^2}\int_{\kappa a}^{\kappa r_0} x\left[\cosh\left(\Psi_{eq}^*\right) - 1\right]dx\end{aligned} \tag{87}$$

where $\Psi_{eq}^* = e\Psi_{eq}/(kT)$ and $x = \kappa r$. In these integrals, $\Psi_{eq}^*(x)$ represent the (dimensionless) electric potential around the colloidal particle when no electric field is applied. We take $\Psi_{eq}^*(\infty) = 0$, and, if the shear plane is at the surface of the particle (as assumed in the present article), we have $\Psi_{eq}^*(x = \kappa a) = e\zeta/(kT)$: the surface potential is equal to the zeta potential.

For the use of an analytical model for fitting experimental data, it is crucial that all parameters are explicitly known and rather easily calculable. Both integrals should therefore be replaced by good analytical approximations. The integrals $I_{c,eq}$ and $I_{n,eq}$ run from κa to κr_0. As the electric potential Ψ_{eq} decays over a characteristic lengthscale $1/\kappa$ to zero, the upper limit can safely be extended to $x = \infty$. From the Poisson-Boltzmann relation we get:

$$\frac{1}{x}\frac{\partial^2}{\partial x^2}\left(x\Psi_{eq}^*\right) = \sinh(\Psi_{eq}^*) \tag{88}$$

Inserting this in eqs.(87) and letting $r_0 \to \infty$ yields:

$$I_{c,eq} = \frac{1}{(\kappa a)^2}\int_{\kappa a}^{\infty}\frac{\partial^2}{\partial x^2}\left(x\Psi_{eq}^*\right)dx = \frac{1}{(\kappa a)^2}\left[\frac{-e\zeta}{kT} - \kappa a\frac{\partial}{\partial x}\left(\Psi_{eq}^*\right)_{x=\kappa a}\right] \tag{89}$$

The integral $I_{c,eq}$ can thus be approximated using existing analytical expressions for the electrokinetic surface charge density q as function of zeta potential. We have

$$\begin{aligned}I_{c,eq} &= \frac{1}{(\kappa a)^2}\left[\frac{-e\zeta}{kT} + \kappa a q^*\right] \\ q^* &= \frac{e}{\kappa\varepsilon_0\varepsilon_e kT}q\end{aligned} \tag{90}$$

where q^* is the dimentionless electrokinetic surface charge. In [17] the analytical expression for q^* used was taken from Loeb et al. [48], which is valid for all zeta potentials and $\kappa a \geq 0.5$. A more accurate

expression was found by Ohshima et al. [1],[49] which holds for $\kappa a \geq 0.1$. This expression leads to the following estimate for $I_{c,eq}$ which holds for $\kappa a \geq 0.1$:

$$I_{c,eq} = \frac{\sqrt{2}}{\kappa a}\left[\cosh\left(\frac{e\zeta}{kT}\right) - 1 + \frac{8}{\kappa a}(\cosh\left(\frac{e\zeta}{2kT}\right) - 1 + \frac{2}{\kappa a}\ln\left(\cosh\left(\frac{e\zeta}{4kT}\right)\right)\right]^{0.5} - \frac{1}{(\kappa a)^2}\frac{e\zeta}{kT} \qquad (91)$$

For large κa this equation simplifies to the $I_{c,eq}$ found using Loeb's approximation. In order to get an estimate for $I_{n,eq}$, we sum the integrals of eqs.(87) to an integral for $I_{c,eq} + I_{n,eq}$:

$$\begin{aligned} I_{c,eq} + I_{n,eq} &= \frac{1}{(\kappa a)^2}\int_{\kappa a}^{1+\kappa a} x\left[\sinh\left(\Psi^*_{eq}\right) - \cosh\left(\Psi^*_{eq}\right) + 1\right] dx \\ &= \frac{1}{(\kappa a)^2}\int_{\kappa a}^{1+\kappa a} x\left(1 - \exp\left(-\Psi^*_{eq}\right)\right) dx \qquad (92) \end{aligned}$$

whereby we have restricted the upper limit to $1 + \kappa a$. The kernel in the last integral can be replaced quite accurately by a linear series expansion around $x = x_a$. This yields:

$$x\left(1 - \exp\left(-\Psi^*_{eq}\right)\right) = -x_a^2 \exp\left(-\Psi^*_{eq}(x_a)\right)\left(\frac{d\Psi^*_{eq}}{dx}\right)_{x=x_a} + x\left[1 + \exp\left(-\Psi^*_{eq}(x_a)\right)\left(x_a\left(\frac{d\Psi^*_{eq}}{dx}\right)_{x_a} - 1\right)\right] \qquad (93)$$

We have explored 2 options for x_a viz. $x_a = \kappa a$ and $x_a = \kappa a + 1/2$. They gave upon integration respectively:

$$\begin{aligned} I^a_{n,eq} + I_{c,eq} &= \frac{1}{2(\kappa a)^2}\left[(1 + 2\kappa a)\left(1 - \exp\left(-\Psi^*_{eq}(\kappa a)\right)\right) + \kappa a\left(\frac{d\Psi^*_{eq}}{dx}\right)_{x=\kappa a}\exp\left(-\Psi^*_{eq}(\kappa a)\right)\right] \\ I^b_{n,eq} + I_{c,eq} &= \frac{1}{2(\kappa a)^2}\left[1 + 2\kappa a - (1 + 2\kappa a)\exp\left(-\Psi^*_{eq}(\kappa a + 1/2)\right)\right] \qquad (94) \end{aligned}$$

Next, an approximation for the potential distribution Ψ^*_{eq} is needed. The easiest to use, that gives good results, is the well-known analytical distribution found for low ζ for which $\sinh(\Psi^*_{eq}) \simeq \Psi^*_{eq}$ over all the distances from the particle [1]:

$$\Psi^*_{eq}(r) = \frac{e\zeta}{kT}\frac{\kappa a}{x}\exp(\kappa a - x) \qquad (95)$$

By inserting this equation into eqs.(94) we obtain:

$$\begin{aligned} I^a_{n,eq} &= -I_{c,eq} + \frac{1}{2(\kappa a)^2}\left[1 + 2\kappa a - \exp\left(-\frac{e\zeta}{kT}\right)\left[1 + 2\kappa a + \frac{e\zeta}{kT}(1 + \kappa a)\right]\right] \\ I^b_{n,eq} &= -I_{c,eq} + \frac{1}{2(\kappa a)^2}\left[(1 + 2\kappa a)\left(1 - \exp\left(\frac{-2\kappa a}{\sqrt{e}(1 + 2\kappa a)}\frac{e\zeta}{kT}\right)\right)\right] \qquad (96) \end{aligned}$$

We found that the expression for $I_{c,eq}$ calculated from the low-zeta expression gave better estimates for $I_{n,eq}$ than the expression of Ohshima. The low-zeta approximation of $I_{c,eq}$ is:

$$I^{low}_{c,eq} = \frac{1}{(\kappa a)^2}\sqrt{(1 + 2\kappa a)\left(\frac{e\zeta}{kT}\right)^2 + 2(\kappa a)^2\left[\cosh\left(\frac{e\zeta}{kT}\right) - 1\right]} - \frac{1}{(\kappa a)^2}\frac{e\zeta}{kT} \qquad (97)$$

It turns out that substitution of $I^{low}_{c,eq}$ in eqs.(96) performs very well down to $\kappa a = 0.5$. However to cover the κa-range down to 0.1 it is possible to combine $I^a_{n,eq}$ and $I^b_{n,eq}$ in the following way:

$$I_{n,eq} = 0.2 I^a_{n,eq} + 0.8 I^b_{n,eq} \qquad (98)$$

which leads to the following estimate for $I_{n,eq}$:

$$I_{n,eq} = -I^{low}_{c,eq} + \frac{1}{10(\kappa a)^2}\left[(1 + 2\kappa a)\left(5 - 4\exp\left(\frac{-2\kappa a}{\sqrt{e}(1 + 2\kappa a)}\frac{e\zeta}{kT}\right)\right) - \exp\left(-\frac{e\zeta}{kT}\right)\left[1 + 2\kappa a + \frac{e\zeta}{kT}(1 + \kappa a)\right]\right] \qquad (99)$$

The two approximations for $I_{c,eq}$ and $I_{n,eq}$ which are in agreement within a few % with the numerical estimation of these integrals are given by eqs.(91,99).
