# Supplementary material for: Compensating for Electrode Polarization in Dielectric Spectroscopy Studies of Colloidal Suspensions: Theoretical Assessment of Existing Methods
Source: Front Chem. 2016 Jul 19;4:30. doi: 10.3389/fchem.2016.00030 (PMC4949231; doi:10.3389/fchem.2016.00030)
Supplement: Supplementary file 2 [file DataSheet2.pdf]

Supplementary material 2: Mathematical derivations

In this appendix, the mathematical derivations to obtain eqs.(167,192) given below are described. Two electrode geometries considered are sketched in Fig.(2.1).

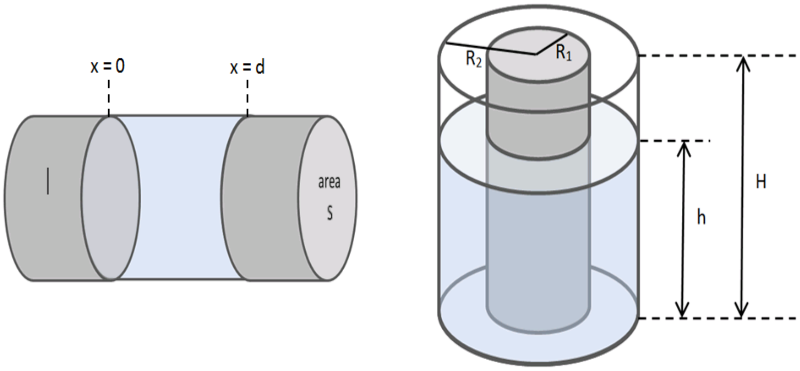

Fig.(2.1): Schematic representation of the two geometries investigated: planar electrodes (left) and cylindrical electrodes (right).

The coordinate in the direction perpendicular to the electrodes will be noted r and the associated unit vector $\mathbf{e}_r$. The liquid between the electrodes consists of an electrolyte solution of known concentration. An alternating voltage is imposed on the electrodes. In the absence of an applied voltage the electrodes are uncharged. No charge transfer is assumed at the electrodes. These "ideally polarizable electrodes" can therefore be considered as capacitor plates.

The set of equations

The flux of positive and negative ions between the electrodes can be described by the Nernst-Planck equation

$$\mathbf{J}_i = -D_i \left(n_i \frac{z_i e}{kT} \boldsymbol{\nabla} \Psi + \boldsymbol{\nabla} n_i \right) \tag{100}$$

Here n_i is the number of ions of type i, and of valence z_i, per unit of volume with $i = +, -$. Ψ is the electric potential. Furthermore, D_i is the ionic diffusion coefficient. We will not consider the influence of ions j on the flux of ions i, which implies that we approximate the conductivity by its value at infinite dilution. We will only consider strong electrolytes and low salt concentrations, in which case this approximation is justified. The conservation of ions gives

$$\frac{\partial n_i}{\partial t} + \boldsymbol{\nabla}.\, \mathbf{J}_i = 0 \tag{101}$$

The charge density is related to Ψ via Poisson's equation:

$$\Delta \Psi \equiv \boldsymbol{\nabla}^2 \Psi = \frac{-1}{\varepsilon_e \varepsilon_0} \sum_i z_i e n_i \tag{102}$$

Because of the electro-neutrality of the salt we have

$$\sum_i \nu_i z_i = 0 \tag{103}$$

where the $\nu_i' s$ are the stoichiometric coefficients of the resulting ions.

An oscillating voltage difference $V_0 \exp(i\omega t)$, where ω is the angular frequency, is applied between the two electrodes. We assume that the electric field is low enough to consider that the perturbations of the various quantities ($\delta\Psi^*$ for the electric potential, δn_i^* for the ionic concentrations) with respect to equilibrium varies linearly with the field. Hence, they are proportional to $\exp(i\omega t)$. This exponential factor will, for ease of notation, further be suppressed by the introduction of the variables $\delta\Psi, \delta n_i$. The subscript "eq" stands for "equilibrium values in the absence of applied field". We therefore have $n_{i,eq} = \nu_i n_\infty$, Ψ_{eq} being constant (and taken to be zero):

$$\begin{aligned} n_i &= n_{i,eq} + \delta n_i^* \exp(i\omega t) \\ &= \nu_i n_\infty + \delta n_i \\ \Psi &= \Psi_{eq} + \delta\Psi^* \exp(i\omega t) \\ &= \delta\Psi \end{aligned} \tag{104}$$

The periodic variables δn_i and $\delta\Psi$ are to be found by solving the equations introduced above. Neglecting non linear terms in eqs.(100,101,102) leads to the following matrix relation [12, 13]:

$$\Delta \begin{pmatrix} \delta n_+ \\ \delta n_- \end{pmatrix} = \begin{bmatrix} i\omega D_+^{-1} + \kappa_0^2 \nu_+ z_+^2 & \kappa_0^2 \nu_+ z_+ z_- \\ \kappa_0^2 \nu_- z_- z_+ & i\omega D_-^{-1} + \kappa_0^2 \nu_- z_-^2 \end{bmatrix} \begin{pmatrix} \delta n_+ \\ \delta n_- \end{pmatrix} \tag{105}$$

with $\kappa_0^2 = e^2 n_\infty \left(\epsilon_0 \epsilon_1 kT\right)^{-1}$ and $\kappa^2 = \kappa_0^2 \sum \nu_i z_i^2$ where κ^{-1} is the Debye screening length. The eigenvalues λ_n^2 and λ_c^2 of the matrix are found to be:

$$\begin{aligned} \lambda_n^2 &= \frac{\kappa^2}{2}(1-R) + \frac{i\omega}{2}\left(\frac{1}{D_+} + \frac{1}{D_-}\right) \quad \text{with} \quad \mathrm{Re}\left(\lambda_n\right) \geq 0 \\ &\simeq i\omega \left(\frac{z_+/D_- - z_-/D_+}{z_+ - z_-}\right) \equiv \frac{i\omega}{D_n} \quad \text{for } \ \omega \ll \kappa^2 D \end{aligned} \tag{106}$$

$$\begin{aligned} \lambda_c^2 &= \frac{\kappa^2}{2}(1+R) + \frac{i\omega}{2}\left(\frac{1}{D_+} + \frac{1}{D_-}\right) \quad \text{with} \quad \mathrm{Re}\left(\lambda_c\right) \geq 0 \\ &\simeq \kappa^2 + i\omega \left(\frac{z_+/D_+ - z_-/D_-}{z_+ - z_-}\right) \equiv \kappa^2 + \frac{i\omega}{D_c} \quad \text{for } \ \omega \ll \kappa^2 D \end{aligned} \tag{107}$$

where we define:

$$R = \sqrt{1 - \frac{\omega^2}{\kappa^4}\left(\frac{1}{D_+} - \frac{1}{D_-}\right)^2 + \frac{2i\omega}{\kappa^2}\frac{\nu_+ z_+^2 - \nu_- z_-^2}{\nu_+ z_+^2 + \nu_- z_-^2}\left(\frac{1}{D_+} - \frac{1}{D_-}\right)} \tag{108}$$

One can verify that these relations, useful for the mathematical derivations, obey:

$$\begin{aligned} \lambda_n^2 \lambda_c^2 &= \frac{-\omega^2}{D_+ D_-} + i\omega\kappa^2 \left[\frac{\nu_+ z_+^2}{D_-} + \frac{\nu_- z_-^2}{D_+}\right] \\ \lambda_c^2 + \lambda_n^2 &= \kappa^2 + i\omega\left(\frac{1}{D_+} + \frac{1}{D_-}\right) \end{aligned} \tag{109}$$

The two left eigenvectors $\begin{pmatrix} x_\lambda & y_\lambda \end{pmatrix}$ associated with the eigenvalue λ ($\lambda = \lambda_n$ or $\lambda = \lambda_c$) respect the relation:

$$\begin{pmatrix} x_\lambda & y_\lambda \end{pmatrix} \begin{bmatrix} i\omega D_+^{-1} + \kappa_0^2 \nu_+ z_+^2 - \lambda^2 & \kappa_0^2 \nu_+ z_+ z_- \\ \kappa_0^2 \nu_- z_- z_+ & i\omega D_-^{-1} + \kappa_0^2 \nu_- z_-^2 - \lambda^2 \end{bmatrix} = \begin{pmatrix} 0 & 0 \end{pmatrix} \tag{110}$$

which gives:

$$\begin{aligned} \left(i\omega D_+^{-1} + \kappa_0^2 \nu_+ z_+^2 - \lambda^2\right) x_\lambda + \kappa_0^2 \nu_- z_- z_+ y_\lambda &= 0 \\ \kappa_0^2 \nu_+ z_+ z_- x_\lambda + \left(i\omega D_-^{-1} + \kappa_0^2 \nu_- z_-^2 - \lambda^2\right) y_\lambda &= 0 \end{aligned} \tag{111}$$

If $D_+ \neq D_-$, substraction of these two equations yields:

$$\left(\frac{i\omega}{D_+} + \kappa^2 - \lambda^2\right) x_\lambda = \left(\frac{i\omega}{D_-} + \kappa^2 - \lambda^2\right) y_\lambda \tag{112}$$

From this last equation, it is possible to impose x_λ so as to determine the corresponding y_λ. By multiplying $(\delta n_+, \delta n_-)$ with the left eigenvectors of the matrix, one obtains:

$$\begin{aligned}
\begin{pmatrix} x_\lambda & y_\lambda \end{pmatrix} \Delta \begin{pmatrix} \delta n_+ \\ \delta n_- \end{pmatrix} &= \begin{pmatrix} x_\lambda & y_\lambda \end{pmatrix} \begin{bmatrix} i\omega D_+^{-1} + \kappa_0^2 \nu_+ z_+^2 & \kappa_0^2 \nu_+ z_+ z_- \\ \kappa_0^2 \nu_- z_- z_+ & i\omega D_-^{-1} + \kappa_0^2 \nu_- z_-^2 \end{bmatrix} \begin{pmatrix} \delta n_+ \\ \delta n_- \end{pmatrix} \\
\Delta \begin{pmatrix} x_\lambda & y_\lambda \end{pmatrix} \begin{pmatrix} \delta n_+ \\ \delta n_- \end{pmatrix} &= \lambda^2 \begin{pmatrix} x_\lambda & y_\lambda \end{pmatrix} \begin{pmatrix} \delta n_+ \\ \delta n_- \end{pmatrix}
\end{aligned} \tag{113}$$

One gets two independent solutions of the ion density variations, δn_n and δn_c, which satisfy

$$\begin{aligned}
\delta n_n &= \begin{pmatrix} x_n & y_n \end{pmatrix} \begin{pmatrix} \delta n_+ \\ \delta n_- \end{pmatrix} \\
\delta n_c &= \begin{pmatrix} x_c & y_c \end{pmatrix} \begin{pmatrix} \delta n_+ \\ \delta n_- \end{pmatrix}
\end{aligned} \tag{114}$$

And

$$\Delta \delta n_n = \lambda_n^2 \delta n_n \quad \text{and} \quad \Delta \delta n_c = \lambda_c^2 \delta n_c \tag{115}$$

These last equations will be solved to yield $\delta n_n(r)$ and $\delta n_c(r)$ in the different geometries. From above, we get, in the case that $D_+ \neq D_-$:

$$\begin{aligned}
\delta n_n &= x_n \delta n_+ + y_n \delta n_- \\
\delta n_c &= x_c \delta n_+ + y_c \delta n_-
\end{aligned} \tag{116}$$

with

$$\begin{aligned}
x_n &= 1 + i\omega / \left(\kappa^2 D_-\right) - \lambda_n^2/\kappa^2 \\
y_n &= 1 + i\omega / \left(\kappa^2 D_+\right) - \lambda_n^2/\kappa^2 \\
x_c &= 1 + i\omega / \left(\kappa^2 D_-\right) - \lambda_c^2/\kappa^2 \\
y_c &= 1 + i\omega / \left(\kappa^2 D_+\right) - \lambda_c^2/\kappa^2
\end{aligned} \tag{117}$$

The sought solutions to eqs.(105) are therefore:

$$\begin{aligned}
\delta n_+ &= \frac{1}{A} \left[-y_n \delta n_c + y_c \delta n_n\right] \\
\delta n_- &= \frac{1}{A} \left[x_n \delta n_c - x_c \delta n_n\right]
\end{aligned} \tag{118}$$

with

$$A \equiv y_c x_n - x_c y_n = \frac{i\omega}{\kappa^4} \left(\lambda_c^2 - \lambda_n^2\right) \left(\frac{1}{D_+} - \frac{1}{D_-}\right) \tag{119}$$

By taking the sum of eqs.(118), one gets:

$$\delta n_+ + \delta n_- = \frac{-\kappa^2}{\left(\lambda_c^2 - \lambda_n^2\right)} \left(\delta n_c - \delta n_n\right) \tag{120}$$

In the special case that $D_+ = D_- = D$ we obtain (for any ω):

$$\begin{aligned}
\lambda_n^2 &= \frac{i\omega}{D} \\
\lambda_c^2 &= \kappa^2 + \frac{i\omega}{D}
\end{aligned} \tag{121}$$

The relations between x_λ and y_λ simplify to:

$$\begin{aligned} x_n &= y_n \\ y_c &= \frac{z_-}{z_+} x_c \end{aligned} \tag{122}$$

In the special case where $D_+ = D_- = D$ we obtain, for any frequency:

$$\begin{aligned} \delta n_n &= \delta n_+ + \delta n_- \\ \delta n_c &= \delta n_+ + \delta n_- \left(\frac{z_-}{z_+}\right) \end{aligned} \tag{123}$$

or, equivalently:

$$\begin{aligned} \delta n_- &= \frac{-z_+}{z_+ - z_-} (\delta n_c - \delta n_n) \\ \delta n_+ &= \frac{z_+}{z_+ - z_-} \left(\delta n_c - \frac{z_-}{z_+} \delta n_n\right) \end{aligned} \tag{124}$$

This should be compared to the case that was derived in [12] for $\omega \ll D_\pm \kappa^2$, for which the eigenvectors reduce to first order to

$$\begin{aligned} \delta n_n &= \delta n_+ + \delta n_- \left[1 + \frac{i\omega}{\kappa^2}\left(D_+^{-1} - D_-^{-1}\right)\right] \simeq \delta n_+ + \delta n_- \\ \delta n_c &= \delta n_+ + \delta n_- \left(\frac{z_-}{z_+}\right)\left[1 - \frac{i\omega}{\kappa^2}\left(D_+^{-1} - D_-^{-1}\right)\right] \simeq \delta n_+ + \delta n_- \left(\frac{z_-}{z_+}\right) \end{aligned} \tag{125}$$

From this follows that, when $D_+ = D_- = D$, the solutions found for frequencies such that $\omega \ll D_\pm \kappa^2$ are valid across the whole range of frequencies.

For frequencies $\omega \ll D_\pm \kappa^2$, the eigenvalues reduce to first order to the expressions given in eqs.(106,107) for that limit. The independent solutions δn_n and δn_c decay over characteristic lengths given by $\sqrt{D_n/\omega}$ and κ^{-1} respectively. δn_c therefore decays over a typical Debye length and δn_n over a typical diffusion length. For the frequencies under consideration the diffusion length is generally much larger than the Debye length, $\sqrt{D_n/\omega} >> \kappa^{-1}$. The solution δn_c will therefore be in good approximation zero a few Debye lengths away from the electrodes. In view of this property we will refer to the layers in which δn_c is unequal to zero as the double layers.

The variable $\delta\Psi$ is found from the Poisson equation that can be re-written:

$$\begin{aligned} \Delta\delta\Psi &= A_c \Delta\delta n_c + A_n \Delta\delta n_n \\ &= A_c \lambda_c^2 \delta n_c + A_n \lambda_n^2 \delta n_n \end{aligned} \tag{126}$$

where

$$\begin{aligned} A_c \lambda_c^2 &= \frac{+e}{\varepsilon_e \varepsilon_0 A}\left[(z_+ - z_-)\left(1 - \frac{\lambda_n^2}{\kappa^2}\right) + \frac{i\omega}{\kappa^2}\left(\frac{z_+}{D_+} - \frac{z_-}{D_-}\right)\right] \\ A_n \lambda_n^2 &= \frac{-e}{\varepsilon_e \varepsilon_0 A}\left[(z_+ - z_-)\left(1 - \frac{\lambda_c^2}{\kappa^2}\right) + \frac{i\omega}{\kappa^2}\left(\frac{z_+}{D_+} - \frac{z_-}{D_-}\right)\right] \end{aligned} \tag{127}$$

The solution $\delta\Psi$ of the Poisson equation takes the form

$$\begin{aligned} \delta\Psi &= A_c \delta n_c + \delta\Psi^+ \\ \delta\Psi^+ &= A_n \delta n_n + \delta\Psi^{++} \\ \delta\Psi^{++} &= F f(r) + G \end{aligned} \tag{128}$$

where $\delta\Psi^+$ symbolises the electric potential beyond the diffuse layer of characteristic length λ_c^{-1} and $\delta\Psi^{++}$ the electric potential beyond the diffusion layer of characteristic length λ_n^{-1}. The function $f(r)$

depends on the geometry of the electrodes, and, because of symmetry, only on the variable r. We have, because of electroneutrality in the bulk (far from the electrodes):

$$\Delta\delta\Psi^{++} = 0 \tag{129}$$

and:

$$\boldsymbol{\nabla}\delta\Psi^{++} = F\boldsymbol{\nabla}f = -\mathbf{E} \tag{130}$$

where $\mathbf{E}$ is the applied electric field. The constant F is determined by the boundary conditions and G can be arbitrarily chosen. We chose $f(r)$ such that F has the dimension of an electric field E. The corresponding charge density is

$$\begin{aligned} \delta\rho &= e\left(z_+\delta n_+ + z_-\delta n_-\right) = -\varepsilon_e\varepsilon_0\lambda_c^2 A_c\delta n_c + \delta\rho^+ \\ \delta\rho^+ &= -\varepsilon_e\varepsilon_0\lambda_n^2 A_n\delta n_n \\ \delta\rho^{++} &= 0 \end{aligned} \tag{131}$$

Boundary conditions

At the electrodes we assume that neither charge transfer nor adsorption takes place ("ideal electrodes"). In view of symmetry, the variables will only depend on the coordinate perpendicular to the electrodes (we neglect boundary effects). We have defined this coordinate r in the general case and will now define r_1 and r_2 as the positions of the two electrodes with $d = r_2 - r_1$ being the separation between the electrodes. We therefore have for the ionic flux:

$$J_i(r_1) = J_i(r_2) = 0 \tag{132}$$

Furthermore it follows from Gauss's law that:

$$\left(\boldsymbol{\nabla}\delta\Psi\right)_{r_j}\cdot\mathbf{e}_r = \frac{-q_j}{\varepsilon_0\varepsilon_e} \tag{133}$$

where q_j is the surface charge density induced by the applied voltage difference on electrode j. Using the Nernst-Planck equation for the ionic fluxes one may replace the boundary conditions for the ion fluxes by two equivalent ones. Dividing eq.(132) by D_i and then summing over i one obtains

$$\left(\boldsymbol{\nabla}\sum_i\delta n_i\right)_{r_1} = \left(\boldsymbol{\nabla}\sum_i\delta n_i\right)_{r_2} = \mathbf{0} \tag{134}$$

where we used electroneutrality. Multiplying eq.(132) by ez_i/D_i and then summing over i, using eq.(133), gives:

$$\left(\boldsymbol{\nabla}\delta\rho\right)_{r_j}\cdot\mathbf{e}_r = \kappa^2 q_j \tag{135}$$

From eq.(134), we get after some calculation:

$$\left(\boldsymbol{\nabla}\delta n_c\right)_{r_j} = \left(\boldsymbol{\nabla}\delta n_n\right)_{r_j} \tag{136}$$

Using eq.(135):

$$\left(\boldsymbol{\nabla}\delta n_c\right)_{r_j}\cdot\mathbf{e}_r = \frac{-q_j}{e}\frac{i\omega}{(z_+ - z_-)}\left(\frac{1}{D_+} - \frac{1}{D_-}\right) \tag{137}$$

From the three last equations we deduce that:

$$\left(\boldsymbol{\nabla}\delta\Psi\right)_{r_j}\cdot\mathbf{e}_r = (A_c + A_n)\left(\boldsymbol{\nabla}\delta n_c\right)_{r_j}\cdot\mathbf{e}_r + F\left(\boldsymbol{\nabla}f(r)\right)_{r_j}\cdot\mathbf{e}_r = \frac{-q_j}{\varepsilon_0\varepsilon_e} \tag{138}$$

And therefore:

$$\begin{aligned} F\left(\boldsymbol{\nabla}f(r)\right)_{r_j}\cdot\mathbf{e}_r &= \frac{-q_j}{\varepsilon_0\varepsilon_e}\left[1 - \frac{\kappa^2}{\lambda_c^2}\frac{i\omega}{\lambda_n^2 D_n}\right] \\ &= \frac{-q_j}{\varepsilon_0\varepsilon_e}\left[1 - \frac{\kappa^2}{\lambda_c^2} + \frac{\kappa^2}{\lambda_n^2\lambda_c^2}\left(\lambda_n^2 - \frac{i\omega}{D_n}\right)\right] \end{aligned} \tag{139}$$

One can verify that for $\omega \ll D_{\pm}\kappa^2$ we have up to second order:

$$\lambda_n^2 \simeq \frac{\omega^2}{4\kappa^2}\left(\frac{1}{D_+} - \frac{1}{D_-}\right)^2 + \frac{i\omega}{D_n} \tag{140}$$

We define D_0 by

$$D_0 = \frac{z_+ D_+ - z_- D_-}{z_+ - z_-} \tag{141}$$

This implies that for $\omega \ll D_{\pm}\kappa^2$:

$$\begin{aligned} F\left(\boldsymbol{\nabla} f\left(r\right)\right)_{r_j} \cdot \mathbf{e}_r &\simeq \frac{-q_j}{\varepsilon_0 \varepsilon_e}\frac{i\omega}{\kappa^2 D_0}\left(1 - \left(\frac{z_+ + z_-}{z_+ - z_-}\right)^2 \frac{D_+ - D_-}{4D_+ D_-}\right) \\ &\simeq \frac{-q_j}{\varepsilon_0 \varepsilon_e}\frac{i\omega}{\kappa^2 D_0} \end{aligned} \tag{142}$$

The second line becomes an equality in the case of binary electrolytes. For non-binary electrolytes it corresponds to a difference of less than 1/1000 in all usual cases, which is therefore not measurable. For $\omega \gg D_{\pm}\kappa^2$ we have

$$R \simeq i\frac{\omega}{\kappa^2}\left(\frac{1}{D_+} - \frac{1}{D_-}\right) \tag{143}$$

and:

$$\begin{aligned} \lambda_n^2 &\simeq \frac{\kappa^2}{2} + i\frac{\omega}{D_-} \simeq i\frac{\omega}{D_-} \\ \lambda_c^2 &\simeq \frac{\kappa^2}{2} + i\frac{\omega}{D_+} \simeq i\frac{\omega}{D_+} \end{aligned} \tag{144}$$

From which we get:

$$F\left(\boldsymbol{\nabla} f\left(r\right)\right)_{r_j} \cdot \mathbf{e}_r \simeq \frac{-q_j}{\varepsilon_0 \varepsilon_e}\left[1 + \frac{i\kappa^2 D_0}{\omega}\right] \tag{145}$$

Link between the complex permittivity and complex impedance of the electrolyte solution

The electric field at any position in the cell, for any geometry, can be evaluated from:

$$\mathbf{E} = -\boldsymbol{\nabla}\delta\Psi = -A_c\boldsymbol{\nabla}\delta n_c - A_n\boldsymbol{\nabla}\delta n_n - F\boldsymbol{\nabla} f\left(\mathbf{r}\right) \tag{146}$$

and the difference of electric potential at the electrodes can be evaluated by:

$$\begin{aligned} V_0 &= \delta\Psi(r_2) - \delta\Psi(r_1) \\ &= A_n\left[\delta n_n(r_2) - \delta n_n(r_1)\right] + A_c\left[\delta n_c(r_2) - \delta n_c(r_1)\right] + F\left[f\left(r_2\right) - f\left(r_1\right)\right] \end{aligned} \tag{147}$$

Moreover, the electric field at the electrodes is linked to the applied potential by (for all geometries)

$$E\left(r_1\right) = E\left(r_2\right) = \frac{V_0}{d} \tag{148}$$

From the difference in electric potential V_0, a "measured" complex dielectric permittivity $\widetilde{\varepsilon}_c$ (or alternatively a measured complex conductivity $\widetilde{K}_c$, using the definition $i\omega\varepsilon_0\widetilde{\varepsilon}_c \equiv \widetilde{K}_c$) can be related to a measured impedance $\widetilde{Z}_c$ by:

$$V_0 \equiv \frac{q_0 d}{\varepsilon_0 \widetilde{\varepsilon}_c} = \frac{q_0 i\omega d}{\widetilde{K}_c} \equiv \widetilde{Z}_c \widetilde{I} \tag{149}$$

where d is the spacing between electrodes and q_0 is a surface charge density. This surface charge density is the surface charge density on each electrode for planar electrodes, but not in the case of cylindrical

electrodes. The expression for the electric current $\widetilde{I}$ is given in the Supplementary material 3 in the case of planar electrodes. Moreover, the relation between the surface charge density q_0 and the charge on each electrode Q depends on the geometry of the electrode. The relations between q_0 and Q are given in eqs.(155,189) for planar and cylindrical coordinates. This geometrical factor is at the origin of the "cell constant" C between measured impedance $\widetilde{Z}_c$ and measured conductance $\widetilde{K}_c$:

$$1/\widetilde{Z}_c = C\widetilde{K}_c$$

where

$$\begin{aligned} C &= \frac{S}{d} \quad \text{(planar electrodes)} \\ C &= 2\pi h \quad \text{(cylindrical electrodes)} \end{aligned} \tag{150}$$

The next subsections detail the derivations for the case of planar and cylindrical electrodes.

Planar electrodes

For symmetry reasons, we take Cartesian coordinates and define $r = x$ as the relevant space variable. The two planar electrodes are located at $x = 0$ and $x = d$. The solutions of eq.(115) are

$$\begin{aligned} \delta n_n &= C_{n,0} \exp\left(-\lambda_n x\right) + C_{n,d} \exp\left(\lambda_n \left(x - d\right)\right) \\ \delta n_c &= C_{c,0} \exp\left(-\lambda_c x\right) + C_{c,d} \exp\left(\lambda_c \left(x - d\right)\right) \end{aligned} \tag{151}$$

where $C_{n,0}$, $C_{n,d}$, $C_{c,0}$ and $C_{c,d}$ are constants to be determined. The solution of the Poisson equation has the form

$$\begin{aligned} \delta\Psi &= A_c \delta n_c + \delta\Psi^+ \\ \delta\Psi^+ &= A_n \delta n_n + Fx + G \end{aligned} \tag{152}$$

The first boundary condition, for blocking electrodes, reads:

$$(J_i)_{x=0} = (J_i)_{x=d} = 0 \tag{153}$$

The second one follows from Gauss:

$$\left(\frac{\partial \delta\Psi}{\partial x}\right)_{x=0} = \left(\frac{\partial \delta\Psi}{\partial x}\right)_{x=d} = \frac{-q_0}{\varepsilon_0 \varepsilon_e} \tag{154}$$

where $q_0 = q_{x=0} = q_{x=d}$ is the charge density on each electrode:

$$q_0 = \frac{Q}{S} \tag{155}$$

The absolute charge on each electrode is $Q = |Q|$ and S the area of each electrode. Furthemore

$$\left(\frac{\partial \delta\rho}{\partial x}\right)_{x=0} = \left(\frac{\partial \delta\rho}{\partial x}\right)_{x=d} = \kappa^2 q_0 \tag{156}$$

From the two last equations, it follows that

$$\begin{aligned} C_n &= C_{n,0} = -C_{n,d} \\ C_c &= C_{c,0} = -C_{c,d} \end{aligned} \tag{157}$$

while

$$F = \frac{-q_0}{\varepsilon_0 \varepsilon_e} \left[1 - \frac{\kappa^2}{\lambda_c^2} \frac{i\omega}{\lambda_n^2 D_n}\right] \tag{158}$$

We have demonstrated that

$$\begin{aligned}\left(\frac{\partial\delta n_c}{\partial x}\right)_{x=0} &= \left(\frac{\partial\delta n_n}{\partial x}\right)_{x=0}\\ \left(\frac{\partial\delta n_c}{\partial x}\right)_{x=0} &= \frac{-q_0}{e}\frac{i\omega}{(z_+-z_-)}\left(\frac{1}{D_+}-\frac{1}{D_-}\right) \qquad (159)\end{aligned}$$

From which we deduce:

$$\begin{aligned}C_c &= \frac{\kappa^2 q_0}{e\lambda_c}\frac{i\omega}{\kappa^2(z_+-z_-)}\left(\frac{1}{D_+}-\frac{1}{D_-}\right)\frac{1}{[1+\exp(-\lambda_c d)]}\\ &= \frac{\kappa^2 q_0}{e\lambda_c}\frac{i\omega}{\kappa^2(z_+-z_-)}\left(\frac{1}{D_+}-\frac{1}{D_-}\right) \quad \text{for} \quad |\lambda_c d| >> 1 \qquad (160)\\ C_n &= \frac{\lambda_c}{\lambda_n}C_{c,0}\frac{[1+\exp(-\lambda_c d)]}{[1+\exp(-\lambda_n d)]} \qquad (161)\\ &= \frac{\lambda_c}{\lambda_n}C_c \quad \text{for} \quad |\lambda_n d| >> 1 \qquad (162)\end{aligned}$$

Note that generally we can assume that the electrodes are several mm apart ($|\lambda_c d| >> |\lambda_n d| >> 1$). We finally get:

$$\begin{aligned}A_c C_c &\simeq \frac{q_0}{\varepsilon_e\varepsilon_0\lambda_c}\frac{\kappa^2}{\lambda_c^2}\frac{1}{[1+\exp(-\lambda_c d)]}\left[\frac{(\kappa^2-\lambda_n^2)}{(\lambda_c^2-\lambda_n^2)}+\frac{i\omega}{D_c(\lambda_c^2-\lambda_n^2)}\right]\\ &\simeq \frac{q_0}{\varepsilon_e\varepsilon_0\lambda_c}\frac{\kappa^2}{\lambda_c^2} \quad \text{for} \;\; \omega \ll \kappa^2 D \quad \text{and} \quad |\lambda_c d| >> 1\\ A_n C_n &\simeq \frac{-q_0}{\varepsilon_e\varepsilon_0\lambda_n}\frac{\kappa^2}{\lambda_n^2}\frac{1}{[1+\exp(-\lambda_n d)]}\left[\frac{(\kappa^2-\lambda_c^2)}{(\lambda_c^2-\lambda_n^2)}+\frac{i\omega}{D_c(\lambda_c^2-\lambda_n^2)}\right]\\ &\simeq 0 \quad \text{for} \;\; \omega \ll \kappa^2 D \quad \text{and} \quad |\lambda_n d| >> 1 \qquad (163)\end{aligned}$$

where D_c is given by eq.(107). The applied potential difference between the electrodes can be written as:

$$\begin{aligned}V_0 &\equiv \delta\Psi(d)-\delta\Psi(0) = -2A_cC_c-2A_nC_n+Fd\\ &= \frac{-q_0 d}{\varepsilon_0\varepsilon_e}+C_c\left[A_c(-2+\lambda_c d)+\frac{\lambda_c}{\lambda_n}A_n(-2+\lambda_n d)\right]\\ &= \frac{-q_0 d}{\varepsilon_0\varepsilon_e}+\frac{-q_0 d}{\varepsilon_e\varepsilon_0}\frac{1}{z_+-z_-}\frac{\kappa^4}{(\lambda_c^2-\lambda_n^2)}\times\\ &\quad \left[\frac{-z_+y_n+z_-x_n}{\lambda_c^2}\left(\frac{-2}{\lambda_c d}+1\right)+\frac{z_+y_c-z_-x_c}{\lambda_n^2}\left(\frac{-2}{\lambda_n d}+1\right)\right] \qquad (164)\end{aligned}$$

Defining the "measured" complex dielectric permittivity $\widetilde{\varepsilon}_c$ by

$$V_0 \equiv \frac{q_0 d}{\varepsilon_0\widetilde{\varepsilon}_c} = \frac{d}{S}\frac{1}{i\omega\varepsilon_0\widetilde{\varepsilon}_c}\widetilde{I} = \frac{d}{S}\frac{1}{\widetilde{K}_c}\widetilde{I} \qquad (165)$$

from which we get the cell constant:

$$C = \frac{S}{d} \qquad (166)$$

We obtain:

$$\widetilde{\varepsilon}_c(\omega) = \varepsilon_e/\left[1-\frac{\kappa^4}{(\lambda_c^2-\lambda_n^2)}\left[\left(1+\frac{i\omega}{\kappa^2 D_c}-\frac{\lambda_n^2}{\kappa^2}\right)\frac{1}{\lambda_c^2}\left[1-\frac{2}{\lambda_c d}\right]-\left(1+\frac{i\omega}{\kappa^2 D_c}-\frac{\lambda_c^2}{\kappa^2}\right)\frac{1}{\lambda_n^2}\left[1-\frac{2}{\lambda_n d}\right]\right]\right] \qquad (167)$$

Cylindrical electrodes

For symmetry reasons, cylindrical coordinates are chosen and we define r as the relevant space variable. The two cylindrical electrodes have the same central axis and the same height h, long enough to neglect end effects. The inner cylinder has a radius R_1 and the outer cylinder a radius R_2, with $(R_2 - R_1) = d$ is the electrode spacing. Eqs.(115) in cylindrical coordinates become:

$$\begin{aligned}
(\lambda_n r)^2 \frac{\partial^2 \delta n_n}{\partial (\lambda_n r)^2} + (\lambda_n r) \frac{\partial \delta n_n}{\partial (\lambda_n r)} - (\lambda_n r)^2 \delta n_n &= 0 \\
(\lambda_c r)^2 \frac{\partial^2 \delta n_c}{\partial (\lambda_c r)^2} + (\lambda_c r) \frac{\partial \delta n_c}{\partial (\lambda_c r)} - (\lambda_c r)^2 \delta n_c &= 0
\end{aligned} \tag{168}$$

The solutions of eq.(168) are given by the order zero modified Bessel functions of first kind I_0 and of second kind K_0, and therefore

$$\begin{aligned}
\delta n_n &= C_n^1 K_0(\lambda_n r) + C_n^2 I_0(\lambda_n r) \\
\delta n_c &= C_c^1 K_0(\lambda_c r) + C_c^2 I_0(\lambda_c r)
\end{aligned} \tag{169}$$

where $C_n^{1,2}$ and $C_c^{1,2}$ are constants to be determined. The solution of the Poisson equation has the form

$$\begin{aligned}
\delta\Psi &= A_c \delta n_c + \delta\Psi^+ \\
\delta\Psi^+ &= A_n \delta n_n + F \ln(r) + G
\end{aligned} \tag{170}$$

The first boundary condition for blocking electrodes reads:

$$J_i = 0 \quad \text{in } r = R_i \text{ with } i = 1, 2 \tag{171}$$

The second one follows from Gauss:

$$\left(\frac{\partial \delta\Psi}{\partial r} \right)_{r=R_i} = \frac{-Q}{2\pi\varepsilon_0\varepsilon_e h R_i} \tag{172}$$

where $Q = |Q|$ is the absolute charge on one or the other electrode. The surface charge density on each electrode is defined by:

$$q_i = \frac{Q}{2\pi h R_i} \tag{173}$$

From eqs.(137,136), we get

$$\begin{aligned}
R_1 \left(\frac{\partial \delta n_c}{\partial r} \right)_{R_1} &= R_2 \left(\frac{\partial \delta n_c}{\partial r} \right)_{R_2} \\
R_1 \left(\frac{\partial \delta n_n}{\partial r} \right)_{R_1} &= R_2 \left(\frac{\partial \delta n_n}{\partial r} \right)_{R_2}
\end{aligned} \tag{174}$$

From the derivatives of the modified Bessel functions of order zero (giving modified Bessel functions of order one):

$$\begin{aligned}
I_0' &= I_1 \\
K_0' &= -K_1
\end{aligned} \tag{175}$$

we get:

$$\begin{aligned}
\left(\frac{\partial \delta n_c}{\partial r} \right) &= -\lambda_c C_c^1 K_1(\lambda_c r) + \lambda_c C_c^2 I_1(\lambda_c r) \\
\left(\frac{\partial \delta n_n}{\partial r} \right) &= -\lambda_n C_n^1 K_1(\lambda_n r) + \lambda_n C_n^2 I_1(\lambda_n r)
\end{aligned} \tag{176}$$

And:

$$\begin{aligned} C_c^2 &= \frac{R_2 K_1(\lambda_c R_2) - R_1 K_1(\lambda_c R_1)}{R_2 I_1(\lambda_c R_2) - R_1 I_1(\lambda_c R_1)} C_c^1 \\ C_n^2 &= \frac{R_2 K_1(\lambda_n R_2) - R_1 K_1(\lambda_n R_1)}{R_2 I_1(\lambda_n R_2) - R_1 I_1(\lambda_n R_1)} C_n^1 \end{aligned} \tag{177}$$

From eq.(137), we get

$$-C_c^1 K_1(\lambda_c R_i) + C_c^2 I_1(\lambda_c R_i) = \frac{-Q}{2\pi R_i \lambda_c he} \frac{i\omega}{(z_+ - z_-)} \left(\frac{1}{D_+} - \frac{1}{D_-} \right) \tag{178}$$

One can verify that this leads to:

$$C_c^1 \left[K_1(\lambda_c R_2) I_1(\lambda_c R_1) - K_1(\lambda_c R_1) I_1(\lambda_c R_2) \right] = \frac{-Q}{2\pi R_1 R_2 \lambda_c he} \frac{i\omega}{(z_+ - z_-)} \left(\frac{1}{D_+} - \frac{1}{D_-} \right) \tag{179}$$

From eq.(136),

$$\lambda_n C_n^1 = \lambda_c C_c^1 \frac{[R_2 I_1(\lambda_n R_2) - R_1 I_1(\lambda_n R_1)]}{[R_2 I_1(\lambda_c R_2) - R_1 I_1(\lambda_c R_1)]} \frac{[K_1(\lambda_c R_2) I_1(\lambda_c R_1) - K_1(\lambda_c R_1) I_1(\lambda_c R_2)]}{[K_1(\lambda_n R_2) I_1(\lambda_n R_1) - K_1(\lambda_n R_1) I_1(\lambda_n R_2)]} \tag{180}$$

And finally from

$$f(r) = \ln(r) \tag{181}$$

we find:

$$F = \frac{-Q}{2\pi\varepsilon_e\varepsilon_0 h} \left[1 - \frac{\kappa^2}{\lambda_c^2} \frac{i\omega}{\lambda_n^2 D_n} \right] \tag{182}$$

Therefore the applied potential difference betwen the electrodes can be written:

$$\begin{aligned} V_0 &= A_n \left[C_n^1 \left(K_0(\lambda_n R_2) - K_0(\lambda_n R_1) \right) + C_n^2 \left(I_0(\lambda_n R_2) - I_0(\lambda_n R_1) \right) \right] \\ &\quad + A_c \left[C_c^1 \left(K_0(\lambda_c R_2) - K_0(\lambda_c R_1) \right) + C_c^2 \left(I_0(\lambda_c R_2) - I_0(\lambda_c R_1) \right) \right] \\ &\quad + \frac{-1}{\varepsilon_0 \varepsilon_e} \frac{Q}{2\pi h} \left[1 - \frac{\kappa^2}{\lambda_c^2} \frac{i\omega}{\lambda_n^2 D_n} \right] \ln\left(\frac{R_2}{R_1} \right) \end{aligned} \tag{183}$$

Under experimental conditions considered, κ^{-1} is of the order of nanometers therefore the condition $|\lambda_c R_i| >> 1$ holds if $R_i >> 10^{-9} m$. Moreover $D_\pm$ is of the order of 10^{-9} m^2/s and $f > 1000$ Hz implying that $|\lambda_n R_i| >> 1$ if $R_i >> 10^{-3} m$. As R_i is of the order of millimeters both conditions are satisfied. The relations relating the Bessel functions:

$$\begin{aligned} I_0(z) &\simeq I_1(z) \simeq \exp(z)/\sqrt{2\pi z} \quad \text{for } |z| \text{ large and } |\arg(z)| < \pi/2 \\ K_0(z) &\simeq K_1(z) \simeq \exp(-z)\sqrt{\pi/(2z)} \quad \text{for } |z| \text{ large and } |\arg(z)| < 3\pi/2 \end{aligned} \tag{184}$$

enable us to write:

$$\begin{aligned} C_c^2 &\simeq \frac{-R_1 K_0(\lambda_c R_1)}{R_2 I_0(\lambda_c R_2)} C_c^1 \\ C_n^2 &\simeq \frac{-R_1 K_0(\lambda_n R_1)}{R_2 I_0(\lambda_n R_2)} C_n^1 \end{aligned} \tag{185}$$

The relation between C_n^1 and C_c^1 becomes

$$\lambda_n C_n^1 = \lambda_c C_c^1 \frac{K_0(\lambda_c R_1)}{K_0(\lambda_n R_1)} \tag{186}$$

and leads to:

$$\begin{aligned} C_c^1 &= \frac{Q}{2\pi\lambda_c heR_1K_0(\lambda_cR_1)}\frac{i\omega}{(z_+ - z_-)}\left(\frac{1}{D_+}-\frac{1}{D_-}\right) \\ C_n^1 &= \frac{Q}{2\pi\lambda_n heR_1K_0(\lambda_nR_1)}\frac{i\omega}{(z_+ - z_-)}\left(\frac{1}{D_+}-\frac{1}{D_-}\right) \end{aligned} \tag{187}$$

The applied potential difference between the electrodes can be rewritten as:

$$V_0 = \frac{-Q}{2\pi\varepsilon_e\varepsilon_0 h}\left[\left(1-\frac{\kappa^2}{\lambda_c^2}\frac{i\omega}{\lambda_n^2 D_n}\right)\ln\left(\frac{R_2}{R_1}\right)+\frac{\kappa^2}{\left(\lambda_c^2-\lambda_n^2\right)}\left[\frac{1}{\lambda_c^3}\left(\kappa^2-\lambda_n^2+\frac{i\omega}{D_c}\right)-\frac{1}{\lambda_n^3}\left(\kappa^2-\lambda_c^2+\frac{i\omega}{D_c}\right)\right]\left(R_1^{-1}+R_2^{-1}\right)\right]$$

Defining the "measured" complex dielectric permittivity $\widetilde{\varepsilon}_c$ by

$$V_0 \equiv \frac{q_0 d}{\varepsilon_0\widetilde{\varepsilon}_c} = \frac{1}{C}\frac{\widetilde{I}}{\widetilde{K}_c} \tag{188}$$

where we have defined the surface charge density q_0 by:

$$q_0 = \frac{Q}{2\pi dh} \tag{189}$$

From which we get the cell constant:

$$C = 2\pi h \tag{190}$$

Note that this surface charge density is related to the surface density on each electrodes by

$$\frac{q_1 - q_2}{q_0} = \frac{d^2}{R_1R_2} \tag{191}$$

We obtain:

$$\widetilde{\varepsilon}_c = \varepsilon_e/\left[\left(1-\frac{\kappa^2}{\lambda_c^2}\frac{i\omega}{\lambda_n^2 D_n}\right)\ln\left(\frac{R_2}{R_1}\right)+\frac{\kappa^2}{\left(\lambda_c^2-\lambda_n^2\right)}\left[\frac{1}{\lambda_c^3}\left(\kappa^2-\lambda_n^2+\frac{i\omega}{D_c}\right)-\frac{1}{\lambda_n^3}\left(\kappa^2-\lambda_c^2+\frac{i\omega}{D_c}\right)\right]\left(R_1^{-1}+R_2^{-1}\right)\right] \tag{192}$$

One can verify that for frequencies such that $\omega \ll D_\pm\kappa^2$, this expression corresponds to the expression found in [13], which is also valid for all types of electrolytes but for frequencies restricted to $\omega \ll D_\pm\kappa^2$. One can similarly calculate the electric field $E = -\delta\Psi(r)/\delta r$ in the solution for any r. It is found that beyond the diffuse layer, at positions r larger than $\left|\lambda_n^{-1}\right|$ from both electrodes:

$$\widetilde{E}(r) \simeq \frac{-F}{r} = \frac{Q}{2\pi h\varepsilon_e\varepsilon_0 r}\frac{i\omega}{\kappa^2 D_0} \tag{193}$$

For spacings such that $R_2 - R_1 = d << R_1$ one may take $r \simeq R_1$ and defining a surface charge density $\sigma_0 \approx 2\pi R_1 lQ \approx 2\pi R_2 lQ$, we recover an expression similar to the one found in the case of planar electrodes, and that $E(r)$ is independent of the position r:

$$\widetilde{E} \simeq \frac{\sigma_0}{\varepsilon_e\varepsilon_0}\frac{i\omega}{\kappa^2 D_0} \tag{194}$$

In the special case where $D_+ = D_- = D$, eq.(192) reduces to:

$$\widetilde{\varepsilon}_{c,e}(\omega) = \varepsilon_e/\left[\left(1-\frac{\kappa^2}{\lambda_c^2}\right)\ln\left(\frac{R_2}{R_1}\right)+\frac{\kappa^2}{\lambda_c^3}\left(R_1^{-1}+R_2^{-1}\right)\right] \tag{195}$$

From the analysis of eq.(192), two important relaxations frequencies are found:

$$\begin{aligned} \omega_C &= \kappa D_0\left(R_1^{-1}+R_2^{-1}\right) \simeq \kappa D_\pm\left(R_1^{-1}+R_2^{-1}\right) \\ \omega_0 &= D_0\kappa^2 \simeq D_\pm\kappa^2 \end{aligned} \tag{196}$$

The relaxation frequency ω_C is related to the relaxation frequency ω_P found for planar electrodes by:

$$\begin{aligned}\omega_C &= \frac{d}{2}\frac{2R_1+d}{R_1\left(R_1+d\right)}\omega_P \\ &= \frac{d}{R_1}\omega_P \quad \text{if} \quad d \ll R_1 \\ &= \frac{d}{2R_1}\omega_P \quad \text{if} \quad d \gg R_1\end{aligned} \tag{197}$$

As in general $d \simeq R_1$ it follows that in this case $\omega_C \simeq \omega_P$. A third frequency is

$$\omega_H = \frac{\kappa^3 D_0}{R_1^{-1}+R_2^{-1}} = \frac{\kappa}{R_1^{-1}+R_2^{-1}}\omega_0 \gg \omega_0$$

Since $\omega_H \gg \omega_0$ this frequency does not apply to any relaxation. The analysis of eq.(195) as function of frequency enables us to predict the following behaviors:
For $\omega \ll \omega_C$ we get:

$$\begin{aligned}\varepsilon_{c,e} &\simeq \varepsilon_e \frac{\kappa}{\left(R_1^{-1}+R_2^{-1}\right)} \\ K_{c,e} &\simeq \left[\frac{\varepsilon_0\varepsilon_e\omega^2}{D_0}\ln\left(\frac{R_2}{R_1}\right)\right] / \left[\left(R_1^{-1}+R_2^{-1}\right)^2\right] \simeq 0\end{aligned} \tag{198}$$

As for planar electrode geometry, the conductivity $K_{c,e}$ is almost zero for low frequencies because the electrodes are blocking. No charge transfer is possible and the ions are all accumulated close to the electrodes.
For $\omega_P \ll \omega \ll \omega_0$ we get:

$$\begin{aligned}\varepsilon_{c,e}\left(\omega\right) &\simeq \varepsilon_e / \left[\kappa\left(R_1^{-1}+R_2^{-1}\right)^{-1}\left(\frac{\omega}{\kappa^2 D_0}\ln\left(\frac{R_2}{R_1}\right)\right)^2\right] \\ K_{c,e} &\simeq \varepsilon_0\varepsilon_e\kappa^2 D_0 / \left[\ln\left(\frac{R_2}{R_1}\right)\right] \equiv K_e / \left[\ln\left(\frac{R_2}{R_1}\right)\right]\end{aligned} \tag{199}$$

In this frequency range (which is the frequency range of interest for experiments) we note that, contrary to what was found for planar electrodes, $K_{c,e}$ is not equal to the conductivity K_e but that there is a constant of proportionality that depends on the cylinders' radii, i.e. $\ln\left(R_2/R_1\right)$ between the two.
For $\omega \gg \omega_0$ we get:

$$\begin{aligned}\varepsilon_{c,e} &\simeq \varepsilon_e / \left[\ln\left(\frac{R_2}{R_1}\right)\right] \\ K_{c,e} &\simeq \varepsilon_0\varepsilon_e\kappa^2 D_0 / \left[\ln\left(\frac{R_2}{R_1}\right)\right] \equiv K_e / \left[\ln\left(\frac{R_2}{R_1}\right)\right]\end{aligned} \tag{200}$$
