# Supplementary material for: Compensating for Electrode Polarization in Dielectric Spectroscopy Studies of Colloidal Suspensions: Theoretical Assessment of Existing Methods
Source: Front Chem. 2016 Jul 19;4:30. doi: 10.3389/fchem.2016.00030 (PMC4949231; doi:10.3389/fchem.2016.00030)
Supplement: Supplementary file 3 [file DataSheet3.pdf]

Supplementary material 3: Equivalent circuit derivations

We will briefly describe how we obtained the equivalence between the analytical solution and the equivalent circuit. A schematic representation of the equivalent circuit is given in Fig.(3.1).

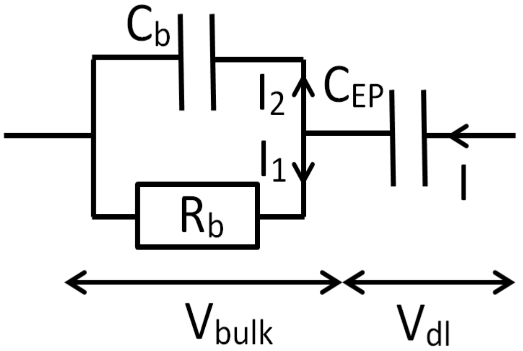

Fig.(3.1): representation of the equivalent circuit

The relations for the current I going through the electrodes and the applied electric voltage V_0 across the cell are:

$$\begin{aligned} I &= I_1 + I_2 \\ V_0 &= V_{bulk} + V_{dl} \end{aligned} \tag{201}$$

The total equivalent circuit (solution and electrode polarization) is represented by

$$\frac{1}{\widetilde{Z}_c} = \frac{i\omega C_{EP}}{1+\omega^2 R_b^2 \left(C_b + C_{EP}\right)^2} \left[1 + \omega^2 R_b^2 C_b \left(C_b + C_{EP}\right) - iR_b C_{EP}\omega\right] \tag{202}$$

We have the following characteristic frequencies:

$$\begin{aligned} \omega_P &= \frac{1}{R_b C_{EP}} \\ \omega_0 &= \frac{1}{R_b C_b} \gg \omega_P \quad \text{as} \quad C_b \ll C_{EP} \\ \omega_{0+P} &= \frac{1}{R_b \left(C_b + C_{EP}\right)} \simeq \omega_P \end{aligned} \tag{203}$$

We have given the frequencies the subscripts introduced in the body of the article, as we will show that these frequencies are indeed equal. The demonstration is done for planar electrodes. The results for cylindrical electrodes can be obtained in the same way.

For very high frequencies such that $\omega \gg \omega_0 \gg \omega_P$ we get

$$\frac{1}{\widetilde{Z}_c} \simeq i\omega C_b + \frac{1}{R_b} \tag{204}$$

So we can make the equivalence:

$$\begin{aligned} \frac{1}{R_b} &= \frac{S}{d} K_e \\ C_b &= C_1 = \frac{S}{d}\varepsilon_e \varepsilon_0 \end{aligned} \tag{205}$$

For very low frequencies for which $\omega_0 \gg \omega_P \gg \omega$ we get

$$\frac{1}{\widetilde{Z}_c} \simeq i\omega C_{EP} + \omega^2 C_{EP} R_b \tag{206}$$

Using the expression obtained for $\widetilde{K}_c$ in this range of frequencies, i.e.

$$\widetilde{K}_c\left(\omega \ll 2\kappa D/d\right) = i\omega\varepsilon_e \frac{\kappa d}{2}\varepsilon_0 + \varepsilon_e\varepsilon_0\left(\frac{\kappa d}{2}\right)^2 \frac{\omega^2}{\kappa^2 D_0} \tag{207}$$

we can put:

$$C_{EP} = \frac{S}{d}\varepsilon_0\varepsilon_e\frac{\kappa d}{2} = \frac{S}{2}\varepsilon_0\varepsilon_e\kappa$$

which leads to

$$\begin{aligned} \omega_P &= \frac{2\kappa D_0}{d} \\ \omega_0 &= \kappa^2 D_0 \end{aligned} \tag{208}$$

as expected.

For the intermediate frequencies of interest, i.e. $\omega_0 \gg \omega \gg \omega_P$ we get:

$$V_0 \simeq V_{bulk} = R_b I_1 \tag{209}$$

Since $I_2 \ll I_1$, we may also put

$$V_0 \simeq R_b I \tag{210}$$

From

$$V_{dl} = \frac{I}{i\omega C_{EP}} \simeq \frac{V_0}{i\omega R_b C_{EP}} \tag{211}$$

we get:

$$V_{bulk} = V_0 - V_{dl} \simeq V_0 - \frac{V_0}{i\omega R_b C_{EP}} \tag{212}$$

This enables us to find that:

$$I_1 = \frac{V_{bulk}}{R_b} \simeq \frac{V_0}{R_b} + \frac{iV_0}{\omega R_b^2 C_{EP}} \tag{213}$$

Moreover, using eq.(209),

$$I_2 = i\omega C_b V_{bulk} \simeq i\omega C_b V_0 \tag{214}$$

The two last equations lead to:

$$I = I_1 + I_2 \simeq \frac{V_0}{R_b}\left[1 + \frac{i}{\omega R_b C_{EP}} + i\omega R_b C_b\right] \tag{215}$$

and therefore:

$$I \simeq \frac{V_0}{R_b}\left[1 + \frac{i\omega_P}{\omega} + \frac{i\omega}{\omega_0}\right] \tag{216}$$

This current is further discussed in Supplementary material 4.
