# Supplementary material for: Compensating for Electrode Polarization in Dielectric Spectroscopy Studies of Colloidal Suspensions: Theoretical Assessment of Existing Methods
Source: Front Chem. 2016 Jul 19;4:30. doi: 10.3389/fchem.2016.00030 (PMC4949231; doi:10.3389/fchem.2016.00030)
Supplement: Supplementary file 4 [file DataSheet4.pdf]

Supplementary material 4: Electric field and current considerations

Instead of working with complex quantities it is of course possible to do the derivations using the in- and out-of-phase parts of the relevant variables. This has been done for instance by Kang et al. [50] for an electrolyte solution in between planar electrodes. In this work, they derive expressions for the bulk electric field. We will compare these expressions to that of the electric field at any position in the cell found in The Supplementary material 2 section, see eq.(146). Kang and al. define the (real) applied electric field as (see eq.(7) in [50]):

$$\begin{aligned}\mathbf{E}_{ext} &= \mathbf{E}_0 \cos(\omega t) \equiv \mathrm{Re}\left(\widetilde{\mathbf{E}}_0\right) \\ \widetilde{\mathbf{E}}_0 &\equiv \mathbf{E}_0 \exp(i\omega t)\end{aligned} \tag{217}$$

where we define $\widetilde{E}_0$ from the oscillating voltage difference $V_0 \exp(i\omega t)$ that is applied across the cell:

$$\widetilde{\mathbf{E}}_0 = \frac{V_0}{d} \exp(i\omega t) \tag{218}$$

where d is the spacing between the electrodes and V_0 the amplitude (chosen to be a real quantity) of the applied voltage difference. The complex permeability of the cell $\widetilde{\varepsilon}_{c,e}$ is defined by eq.(165), and is linked to E_0 and $\widetilde{I}_0$ (the complex current per unit area between the electrodes) by:

$$E_0 \equiv \frac{\widetilde{I}_0}{i\omega\varepsilon_0\widetilde{\varepsilon}_{c,e}} = \frac{\widetilde{q}_0}{\varepsilon_0\widetilde{\varepsilon}_{c,e}} \tag{219}$$

The complex electric charge $\widetilde{q}_0$ (that we now write with a tilde to indicate it is a complex quantity) on the electrodes per unit area is related to the (non-complex) electric charge per unit area q_0 by:

$$q_0 \equiv \mathrm{Re}\left(\widetilde{q}_0 \exp(i\omega t)\right) \tag{220}$$

The complex current is defined by $\widetilde{I} = i\omega\widetilde{q}_0 S$ and the complex current per unit surface by $\widetilde{I}_0 = \widetilde{I}/S$. We define the electric field within the cell, at any position x, by:

$$\begin{aligned}\widetilde{\mathbf{E}}_{cell}(x) &= \widetilde{\mathbf{E}}(x) \exp(i\omega t) \\ \mathbf{E}_{cell}(x) &= \mathrm{Re}\left(\widetilde{\mathbf{E}}(x)\right)\cos(\omega t) - \mathrm{Im}\left(\widetilde{\mathbf{E}}(x)\right)\sin(\omega t)\end{aligned} \tag{221}$$

For the particular case (studied by Kang et al.) of electrolytes where $D_- = D_+ = D$ we obtain from eq.(146) the electric field at any position in the cell, $\widetilde{E}(x)$, for any frequency, by:

$$\begin{aligned}\widetilde{E}(x) &= -\left(\frac{\partial\delta\widetilde{\Psi}}{\partial x}\right) = \widetilde{E}_{dl}(x) + \widetilde{E}_{bulk} \\ \widetilde{E}_{dl}(x) &= \frac{\widetilde{q}_0}{\varepsilon_0\varepsilon_e}\left[\frac{\kappa^2}{\lambda_c^2}\left[\exp(-\lambda_c x) + \exp(\lambda_c(x-d))\right]\right] \\ \widetilde{E}_{bulk} &= \frac{\widetilde{q}_0}{\varepsilon_0\varepsilon_e}\left[1 - \frac{\kappa^2}{\lambda_c^2}\right]\end{aligned} \tag{222}$$

where $\widetilde{E}_{dl}(x)$ represents the contribution to the electric field due to the presence of the double layers and $\widetilde{E}_{bulk}$ the contribution from the bulk, beyond the double layers (or more precisely, a length $\lambda_c^{-1} \simeq \kappa^{-1}$ away from the electrodes. For a general electrolyte, for $\omega \ll \omega_0$, one can see from eq.(146) that it would be a length $\lambda_n^{-1} > \lambda_c^{-1}$ away from the electrodes). Note that the electric field $\widetilde{E}_{bulk}$ does not depend on

x whereas $\widetilde{E}_{dl}$ does. By definition, the electric potential across the cell is given by the sum of the electric potential across the double layer and the bulk:

$$V_0 = \widetilde{V}_{dl} + \widetilde{V}_{bulk} \tag{223}$$

Note that this equality implies that $\mathrm{Im}(\widetilde{V}_{dl}) = -\mathrm{Im}(\widetilde{V}_{bulk})$ since we have imposed that V_0 is a real quantity. From the definition of $\widetilde{I}_0$ we get:

$$\widetilde{E}_{bulk} = \frac{\widetilde{I}_0}{i\omega\varepsilon_0\varepsilon_e}\left[1 - \frac{\kappa^2}{\lambda_c^2}\right] \tag{224}$$

From eq.(219), we get:

$$E_0 = \frac{V_0}{d} \equiv \frac{\widetilde{q}_0}{\varepsilon_0\widetilde{\varepsilon}_{c,e}} = \frac{\widetilde{I}_0}{i\omega\varepsilon_0\widetilde{\varepsilon}_{c,e}} \tag{225}$$

From the last two equations, we obtain:

$$\frac{\widetilde{E}_{bulk}}{E_0} = \frac{\widetilde{\varepsilon}_{c,e}}{\varepsilon_e}\left[1 - \frac{\kappa^2}{\lambda_c^2}\right] \tag{226}$$

One can define:

$$E_{bulk} = \mathrm{Re}\left(\widetilde{E}_{bulk}\exp(i\omega t)\right) \tag{227}$$

implying that:

$$\frac{E_{bulk}}{E_0} = \mathrm{Re}\left(\frac{\widetilde{\varepsilon}_{c,e}}{\varepsilon_e}\left[1 - \frac{\kappa^2}{\lambda_c^2}\right]\exp(i\omega t)\right) \tag{228}$$

This expression is to be compared to the expression found by Kang et al. (their eq.(18)),

$$E_{bulk} = E_0\left[P'_{bulk}\cos(\omega t) + P''_{bulk}\sin(\omega t)\right] \tag{229}$$

from which we deduce that:

$$\begin{aligned} P'_{bulk} &\equiv \mathrm{Re}\left(\frac{\widetilde{\varepsilon}_{c,e}}{\varepsilon_e}\left[1 - \frac{\kappa^2}{\lambda_c^2}\right]\right) \\ P''_{bulk} &\equiv -\mathrm{Im}\left(\frac{\widetilde{\varepsilon}_{c,e}}{\varepsilon_e}\left[1 - \frac{\kappa^2}{\lambda_c^2}\right]\right) \end{aligned} \tag{230}$$

Or in other words:

$$\frac{\widetilde{\varepsilon}_{c,e}}{\varepsilon_e} = \frac{\widetilde{P}_{bulk}}{1 - \kappa^2/\lambda_c^2} \tag{231}$$

where $\widetilde{P}_{bulk} = P'_{bulk} - iP''_{bulk}$. Explicit expressions for P'_{bulk} and P''_{bulk} are given by eqs.(44) in [50], which can be used to derive explicit formulas for $\varepsilon'_{c,e}$ and $\varepsilon''_{c,e}$. However, explicit expressions for the real and imaginary part of $\widetilde{\varepsilon}_{c,e}$ can also be obtained directly from eq.(25):

$$\begin{aligned} \mathrm{Re}\left(\widetilde{\varepsilon}_{c,e}\right) &= \varepsilon'_{c,e} = \varepsilon_e\frac{4\kappa d\omega_n y\left[1 - 2y^2\right] + (\kappa d\omega_n)^2\left(\omega_n^2 + 4y^4\right)}{16y^2\left(1 - \kappa d\omega_n y\right) + (\kappa d\omega_n)^2\left(\omega_n^2 + 4y^4\right)} \\ \mathrm{Im}\left(\widetilde{\varepsilon}_{c,e}\right) &= -\varepsilon''_{c,e} = \varepsilon_e\kappa d\frac{4y\left(\omega_n^2 + 2y^2\right) - \kappa d\omega_n\left(\omega_n^2 + 4y^4\right)}{16y^2\left(1 - \kappa d\omega_n y\right) + (\kappa d\omega_n)^2\left(\omega_n^2 + 4y^4\right)} \end{aligned} \tag{232}$$

in which we have introduced the (non-complex) variables y and ω_n that are related to λ_c by:

$$\begin{aligned} \lambda_c &= \kappa\sqrt{1 + i\omega_n} = \kappa\left(\frac{\omega_n}{2y} + iy\right) \\ \omega_n &= \frac{\omega}{\omega_0} \\ y &= \frac{1}{\sqrt{2}}\sqrt{-1 + \sqrt{1 + \omega_n^2}} \end{aligned} \tag{233}$$

These variables can be linked to the variables used by Kang et al. (see eqs.(36, 45, 46) in [50]) by

$$\begin{aligned}\Lambda &\equiv \omega_n = \frac{\omega}{\omega_0} \\ \Omega &\equiv \omega_n \kappa d = \frac{2\omega}{\omega_P} \\ W &\equiv 2y/\omega_n\end{aligned} \tag{234}$$

From eq.(226) and eq.(25), we get in complex quantities:

$$\frac{\widetilde{E}_{bulk}}{E_0} = \frac{i\Omega}{i\Omega + 2\left(1 + i\omega_n\right)^{-1/2}} \tag{235}$$

For $\omega_n \ll 1$ it is clear that $\left(1 + i\omega_n\right)^{-1/2} \simeq 1$. For $\omega_n \gg 1$ it follows that $\left(1 + i\omega_n\right)^{-1/2} \simeq 0$ and $\Omega \gg 1$. This corresponds to the condition indicated by Kang et al. who state that " In the experiments, the Debye length is very much smaller than the distance L between the electrodes" which can be translated into: $\Omega = \omega_n \kappa d \gg \omega_n$. For all frequencies, one therefore has:

$$\frac{\widetilde{E}_{bulk}}{E_0} \simeq \frac{i\Omega}{i\Omega + 2} \tag{236}$$

leading to

$$\begin{aligned}P'_{bulk} &\simeq \frac{\Omega^2}{4 + \Omega^2} \\ P''_{bulk} &\simeq \frac{-2\Omega}{4 + \Omega^2}\end{aligned} \tag{237}$$

as found by Kang, see their eq.(20). Below their eq.(19), Kang et al. state that "For very large frequencies, P'_{bulk} tends to unity while P''_{bulk} vanishes, that is, the electric field in the bulk of the solution is now equal to the applied field. The double layers at the electrodes have no time to develop for these high frequencies, and therefore do not screen the applied electric field". In fact, one can verify that for all frequencies such that $\omega \gg \omega_p$ (which implies $\Omega \gg 1$):

$$\frac{\widetilde{E}_{bulk}}{E_0} \simeq 1 \tag{238}$$

From the equivalent circuit approach (see Supplementary material 2), a detailed study can be made of the electric field, electric potential and electric current across the whole frequency range. For $\omega \ll \omega_P$ ($\Omega \simeq 0$), we find from eqs.(206, 236):

$$\begin{aligned}\frac{\widetilde{E}_{bulk}}{E_0} &\simeq 0 \\ \widetilde{I} &\simeq iC_{EP}\omega V_0 \left(\simeq 0\right)\end{aligned} \tag{239}$$

Moreover:

$$\begin{aligned}\left|\widetilde{V}_{dl}\right| &= \int_0^d \left|\widetilde{E}_{dl}\left(x\right)\right| dx \simeq V_0 \\ \left|\widetilde{V}_{bulk}\right| &\simeq 0\end{aligned} \tag{240}$$

This implies that for this frequency range, as stated by Kang et al. below their eq.(19), " the bulk response function vanish, that is, the electric field in the bulk solution is completely screened by the double layers at the electrodes".

For $\omega \gg \omega_p$ eqs.(225, 226) give:

$$\begin{aligned}\widetilde{\varepsilon}_{c,e}\left(\omega\right) &= \varepsilon_e / \left[1 - \left(\frac{\kappa}{\lambda_c}\right)^2 \left[1 - \frac{2}{\lambda_c d}\right]\right] \\ \text{with} \quad \lambda_c &= \sqrt{\kappa^2 + i\omega/D}\end{aligned} \tag{241}$$

As $\kappa d \gg 1$ (the electrodes are spaced much more apart than a Debye length), one gets for any frequency,

$$\widetilde{\varepsilon}_{c,e}(\omega) \simeq \varepsilon_e / \left[1 - \left(\frac{\kappa}{\lambda_c}\right)^2\right] \tag{242}$$

Therefore

$$\widetilde{E}_{bulk} = \frac{\widetilde{I}_0}{i\omega\varepsilon_0\varepsilon_e}\left[1 - \frac{\kappa^2}{\lambda_c^2}\right] \simeq \frac{\widetilde{I}_0}{i\omega\varepsilon_0\widetilde{\varepsilon}_{c,e}} = \frac{\widetilde{I}_0}{\widetilde{K}_{c,e}} = E_0 \tag{243}$$

Beyond the diffuse layer, for frequencies higher than ω_p the electric field $\widetilde{E}_{bulk}$ is in good approximation equal to the applied voltage difference divided by the electrode spacing, referred to as "applied electric field" E_0. (Using eq.(236), this is the case when $\Omega \gg 1$). For $\omega_p \ll \omega \ll \omega_b$ one has $\widetilde{K}_{c,e} \simeq K_e$ as $\mathrm{Re}\left(\widetilde{K}_{c,e}\right) \simeq \mathrm{Re}\left(\widetilde{K}_e\right)$, $\mathrm{Re}\left(\widetilde{K}_{c,e}\right) \gg \mathrm{Im}\left(\widetilde{K}_{c,e}\right)$, $\mathrm{Re}\left(\widetilde{K}_e\right) \gg \mathrm{Im}\left(\widetilde{K}_e\right)$ but note that $\mathrm{Im}\left(\widetilde{K}_{c,e}\right) \neq \mathrm{Im}\left(\widetilde{K}_e\right)$. One then obtains:

$$E_0 \simeq E_{bulk} \simeq \frac{I_0}{K_e} \tag{244}$$

When $\omega \gg \omega_b$: $\mathrm{Re}\left(\widetilde{K}_{c,e}\right) \simeq \mathrm{Re}\left(\widetilde{K}_e\right)$ and $\mathrm{Im}(K_{c,e}) \simeq \mathrm{Im}\left(\widetilde{K}_e\right)$ one gets:

$$\begin{aligned}
E_0 &\simeq \widetilde{E}_{bulk} \simeq \frac{\widetilde{I}_0}{\widetilde{K}_e} \\
E_0 &\simeq E_{bulk} \simeq \frac{I_0}{K_e} \quad \text{for} \quad \omega \ll \omega_0 \text{ as } \widetilde{K}_e \simeq K_e \\
E_0 &\simeq E_{bulk} \simeq \frac{\widetilde{I}_0}{i\omega\varepsilon_0\varepsilon_e} \quad \text{for} \quad \omega \gg \omega_0 \text{ as } \widetilde{K}_e \simeq i\omega\varepsilon_0\varepsilon_e
\end{aligned} \tag{245}$$

For $\omega_0 \gg \omega \gg \omega_p$ one gets from eqs.(244, 245) $E_0 \simeq E_{bulk}$ and $\left|\widetilde{E}_{dl}(0)\right| \gg \left|\widetilde{E}_{bulk}\right|$ which means that the surface charges are screened by the double layers. However, the electric potential is not screened by the double layer because:

$$\left|\widetilde{V}_{dl}\right| = \int_0^d \left|\widetilde{E}_{dl}(x)\right| dx \ll \left|\widetilde{E}_{bulk}\right| d = \left|\widetilde{V}_{bulk}\right| \tag{246}$$

From eq.(216), one gets for $\omega_0 \gg \omega \gg \omega_p$

$$I \simeq \frac{V_0}{R_b}\left[1 + \frac{i\omega_P}{\omega} + \frac{i\omega}{\omega_0}\right] \tag{247}$$

The real part of the right-hand side this equation is much larger than its imaginary part which leads to

$$I \simeq \frac{V_0}{R_b} \quad \text{corresponding to} \quad I_0 \simeq \frac{E_0}{K_e} \tag{248}$$

Interestingly, one finds easily from eq.(247) that

$$I \simeq \frac{V_0}{R_b}\left[1 + \frac{i\omega}{\omega_0}\right] \quad \text{corresponding to} \quad I_0 \simeq \frac{E_0}{\widetilde{K}_e} \tag{249}$$

holds when $\omega \gg \sqrt{\omega_P\omega_0} = \omega_b$. The frequency ω_b is the one we found to correspond to the frequency above which the electrode polarization becomes negligible. More precisely, it is the frequency that represents the transition between $\mathrm{Im}\left(\widetilde{K}_{c,e}\right) \neq \mathrm{Im}\left(\widetilde{K}_e\right)$ and $\mathrm{Im}\left(\widetilde{K}_{c,e}\right) \simeq \mathrm{Im}\left(\widetilde{K}_e\right)$.

For $\omega \gg \omega_0$ one still has $E_0 \simeq \widetilde{E}_{bulk}$ but the surface charges are not screened by the double layers. Therefore the electric field in the whole system is given by Gauss' law (using the surface charge $\widetilde{q}_0$), which implies that $\widetilde{E} \simeq \widetilde{E}_{bulk}$ and consequently $\left|\widetilde{E}_{dl}(0)\right| \ll \left|\widetilde{E}_{bulk}\right|$. The double layers do not exist anymore (in the sense that the ions close to the electrodes do not screen the surface charges properly), however, there is still a small ionic density close to the electrodes that is different from the bulk density. This ionic density reaches the bulk ionic density at the characteristic length $\lambda_c^{-1} \simeq \sqrt{D/\omega}$ from the electrodes.
